# Mass Mobilization in the Modern Era: Introducing the Opposition Movements and Groups (OMG) Dataset, 1789–2019

**DOI:** 10.1177/00104140251369330

**Published:** 2025-08-27

**Authors:** Marianne Dahl, Sirianne Dahlum, Hanne Fjelde, Haakon Gjerløw, Carl Henrik Knutsen, Carina Strøm-Sedgwick, Tore Wig

**Affiliations:** 1 6287 Peace Research Institute Oslo (PRIO), Oslo, Norway; 2 University of Oslo, Oslo, Norway; 3 Uppsala University, Uppsala, Sweden

**Keywords:** democratization and regime change, social movements, quantitative methods, non-democratic regimes

## Abstract

The nature and consequences of mass mobilization are core topics in the social sciences. How have mass mobilization movements evolved over time? How do key characteristics of such movements, including their social composition or ideology, influence their ability to overthrow or alter political institutions? We introduce the Opposition Movements and Groups (OMG) dataset, which – due to its unique contents and extensive coverage – will help researchers to better address these and many other questions about mass mobilization movements. OMG includes information on the stated goals, duration, size, tactics, ideology, and social and organizational composition of 1452 mass mobilization movements, globally, from 1789 to 2019. We discuss OMG’s contents, construction, validity and reliability issues, and how it complements existing datasets. We showcase the data, first, by documenting several key trends in movement characteristics since the French Revolution, and, second, by shedding new light on the much-discussed relationship between nonviolent movements and democratization.

## Introduction

Mass mobilization movements have, for centuries, been a driving force of social and political developments, and the topic has been widely studied in political science and historical sociology. The vast scholarship on this subject has focused on the social forces underpinning mass movements, the ideologies and political claims that animate them, and the tactics they employ to achieve their goals. Yet, despite the extensive literature on such movements, we still lack a comprehensive global data source that systematically integrates the social (group) composition of the movements, their ideological profiles, and the tactics they employ in one template. Another issue is that most datasets cover only the 20th and 21st centuries. These shortcomings impede our efforts to address some of the most foundational questions in the historically oriented literature on mass movements and political change, such as: Which social groups have participated in mass movements in different regions and periods of the modern era? Which political ideologies have been more effective in engendering revolutionary change? When do citizens mobilize to promote democracy, and when do they mobilize in favor of authoritarian regimes? Has the effectiveness of protest tactics in promoting political change varied throughout history?

We present a unified data source that will aid researchers in tackling these and similar questions; the Opposition Movements and Groups (OMG) dataset.^1^ The dataset spans 1452 distinct mass mobilization movements from 1789 to 2019 across 151 independent states and colonies. Crucially, it comprises rich information on the social composition, political ideology, immediate political claims, and varied tactics of mass movements. The movements that we record represent sizeable instances of mass collective action, coordinated with the explicit intent to change (or strengthen) the current political regime, alter core institutions or liberties, remove the head of state or government, enhance regional autonomy, promote independence, or otherwise change the state’s territorial composition.^2^ While varying across several dimensions – from longevity to ideological underpinnings to social group composition to the use of violence – they have contributed to shaping the world we live in today.

The introduction of OMG opens up numerous new avenues for theoretical and empirical exploration: First, with its extensive coverage going back to 1789, OMG provides the first systematic cross-country dataset on mass mobilization movements and their characteristics in the early modern historical period. While the temporal scope of the most prominent cross-country dataset, NAVCO (Chenoweth & Stephan, 2011), is impressive – extending back to 1900 – this still leaves about half of “modern history” undocumented. The much longer time-series in OMG enables scholars to investigate the prevalence, characteristics, drivers, and consequences of mass mobilization throughout recent centuries, and explore potential changes (or persistence) in the nature of mass mobilization over time.

Second, our dataset covers a wide range of mass mobilization movements that have not been studied in a comparative, historical fashion before, such as protest movements aiming to push for institutional reforms without overthrowing the regime – including protests with anti-liberal goals, as well as pro-regime protests. Previous data sources typically focus on protest events *or* are limited to “maximalist campaigns” aimed at overthrowing existing regimes or altering the state’s territorial integrity (Beissinger, 2022a; Chenoweth et al., 2017; Chenoweth & Stephan, 2011; Chin, 2017). We know considerably less about the drivers and outcomes of movements with less expansive goals. OMG catalogs campaigns with less expansive demands, such as increased territorial autonomy without seceding from the state, unseating incumbent leaders without pursuing full regime change, or pushing for institutional reforms within the framework of the existing regime.

The latter include calls for more inclusive institutions, such as expanding voting rights or strengthening civil liberties, but OMG also documents movements advocating for less inclusive institutions - in contrast to previous datasets which typically tend to focus on pro-democracy movements. Further, OMG includes *pro-regime movements*, where mobilization occurred to maintain the status quo or further empower the incumbent regime.^3^ In a world where authoritarian sentiments are on the rise – and authoritarian regimes are solidifying their power – it is important to be able to also assess the drivers and consequences of anti-liberal protests and pro-regime protests (in autocracies).

Third, OMG includes more than 100 variables, offering detailed information on characteristics of protest movements that are absent or insufficiently covered in existing datasets, such as movement ideology, social group composition, and organization.^4^ Scholars across different social science disciplines have emphasized the significance of these characteristics and their inter-linkages in shaping key outcomes, including regime change and revolutions (Moore, 1966; Rueschemeyer et al., 1992; Skocpol, 1979). Yet, no existing dataset integrates the broader set of relevant factors necessary for systematically examining these connections within a single framework. To illustrate how OMG’s integration of social composition, ideology, and tactics may facilitate re-assessments of key questions in the literature, we present an empirical application that re-evaluates the relationship between nonviolent (vs. violent) mass mobilization and subsequent democratization. While affirming the overall positive relationship between predominantly nonviolent strategies and democratization, our findings reveal that this relationship is contingent on movements’ social composition, political claims, stated ideology, and the historical context. Specifically, we find that nonviolent movements are conducive to democratization after 1900, but not before. Notably, we find that this is not only driven by movements advocating for regime change: Also movements seeking more modest forms of institutional change are conducive to democratization, which underscores the need to also incorporate these to understand how protest shapes democratization. Furthermore, nonviolent movements with nationalist or conservative ideologies are not linked to democratization. We also show how nonviolent protest only seems conducive to democratization if dominated by students, workers or intellectuals, but not if dominated by the urban middle classes, peasants or military personnel. Hence, *who the protesters are and what they want* seem to matter for the political outcomes of protest - and our dataset opens up fruitful avenues for further exploration along these lines.

Before introducing this application, we will, in sequence, discuss existing datasets and some of the research building on them, OMG’s contents and key innovations, the data collection process and data quality, and several notable – including hitherto undocumented – historical trends in mass mobilization movements across the world.

## Research and Data on Mass Movements

Scholars across different research fields have long recognized the significance of mass mobilization for understanding the development of societies and political regimes. Studies of such movements go beyond the day-to-day workings of political systems or behavior *within* given institutional frameworks. Instead, they consider extra-institutional and more irregular political behavior and events, including protests, revolutions, insurgencies, and other forms of mass mobilization (e.g., Tarrow, 1998; Tilly, 1978). Despite their irregular and fairly infrequent nature, such mass collective action often constitutes important, albeit informal, channels of political influence in many societies. At critical junctures in countries’ histories, they influence how power is distributed by shaping whether institutions die and how (and which) new institutions are born (e.g., Chenoweth & Stephan, 2011).

Hence, social scientists have long been preoccupied with questions such as: Why and when do mass protests (or other forms of contentious collective action) erupt (e.g., Gurr, 1970; Tilly, 1978)? How have their ideologies and composition changed across history, and how does this evolution, in turn, shape societies (e.g., Beissinger, 2022a)? And, under what conditions are mass mobilization movements likely to contribute to policy change, specific institutional changes, or even to more comprehensive outcomes such as democratization (Acemoglu & Robinson, 2006; Rueschemeyer et al., 1992)? In-depth case studies exploring these questions abound, detailing events and processes in one or several countries over limited periods of time (e.g., Beissinger, 2013; Bunce & Wolchik, 2011; Collier, 1999a; Goldstone, 2001; Lohmann, 1994; Wood, 2001). Detailed data from one or a few cases can unveil important and detailed insights about, for instance, the demands or dynamics of particular movements. Yet, it is difficult to know how generalizable insights from these studies are. Likewise, it is difficult to answer general questions about how much contextual factors influence the probability of sustained mass opposition movements occurring and what their typical impacts are. To answer such questions, cross-country time-series data with extensive coverage is indispensable.

### Existing Datasets

Several relevant cross-country datasets on mass mobilization movements have already been collected, as summarized in Table 1. Existing datasets can be divided into event-oriented and campaign-oriented datasets.^5^ Initial cross-national work, for example on the effects of popular mobilization on political transitions, relied on event-data from the Cross-National Time-Series (CNTS) Data Archive (Banks, 2011).^6^ Over the last decade, the number of event datasets has increased rapidly. These fine-grained datasets often encompass daily information on strategies and tactics, making them instrumental in unveiling the micro-mechanisms of mass mobilization. Some record activities of both dissidents and regimes, allowing researchers to study dynamic interactions.^7^ Other datasets focus on events in specific regime types, regions, or locations, and cover them comprehensively.^8^

**Table 1.** Existing Datasets.

Dataset	Units	Coverage	Time-Period	Claims	Min. size	Strategy	*N*
NAVCO 1.3	Campaigns	Global	1900–2019	Maximalist	1000	NV/V	622
MEC	Campaigns	Global	1955–2013	Maximalist	1000	NV	70
Enhanced Major Episodes of Contention	Campaigns	Global	1955–2018	Reformist and maximalist	1000		2101
Revolutionary Episodes	Campaigns	Global	1955–2018	Maximalist			343
Anatomy of Resistance Campaigns (ARC) dataset	Organization-country-years	Africa	1900–2015	Maximalist	1000		3,407
NAVCO 2.1	Campaign-years	Global	1945–2013	Maximalist	1000	NV/V	2,717 campaign-years (384 campaigns)
NAVCO 3.0	Events and campaigns	Global	1945–2019	Maximalist, some reformist	1000	NV/V	604 campaigns, 20,000+ events
NEVER	Campaign-years	Global	1945–2013	Maximalist	1000	NV/V	604 campaigns
Cross-National Time-Series Data Archive	Events	Global	1815–2022		100	NV/V	
Mass Mobilization Dataset	Events	Global	1990–2018	Political claims	50		10,000
ACLED	Events	Global	Varies				
MMAD	Events	Autocracies	2003–2019	Political claims	25	Protest events	
SCAD	Events	Africa and Latin America	1990–2017	Social conict and political disorder	No minimum	NV/V	23,246
UCDP Georeferenced Event Dataset	Events	Global	1989–2021	State-based, non-state, and one-sided violence	>1 fatality	V	2,93,634
European Protest and Coercion Data	Events	Europe	1980–1995	Non-maximalist: Domestic conficts	No minimum	NV	
PolDem-Protest Dataset	Events	Europe	2000–2015	Non-maximalist	No minimum	NV	
SPEED	Events	Global	1946-2013	Demonstrations, strikes, riots, and anti-government protests	100	NV/V	15,857
Brancati Democracy Protests Dataset	Events	Global	1989–2011	Pro-democracy protests	No minimum	NV/V	183 countries
ICEWS	Events	Global	1995–present	Political and social unrest, coups, strikes, and protests	No minimum	Various	Millions

*Note*. This table presents commonly used protest datasets but is not an exhaustive list. It includes cross-country datasets, both global and regional, that are influential in the study of protest movements and events. Country-specific datasets, such as Dynamics of Collective Action in the United States (Earl et al., 2003), and sub-national datasets like the Urban Social Disorder Data (Thomson et al., 2023), are not included.

At the opposite end of the spectrum are campaign-oriented datasets, like OMG. While lacking the fine-grained details found in event-based datasets, they offer the advantages of accounting for the interrelatedness of events within the same movement and recording movement-wide attributes. Analyzing such aggregated data, which typically span wider temporal and geographical scopes, can provide insights into the organization and execution of mass mobilization, along with critical factors contributing to their success or failure.

NAVCO is the landmark dataset by Chenoweth and Stephan (2011), and the two most widely used versions (1.1 & 2.0) are campaign-oriented. NAVCO 1.1 covers 323 campaigns from 1900 to 2006, and NAVCO 2.0 covers 250 campaigns from 1945 to 2006. The most expansive version, NAVCO 1.3, contains information on 622 violent and nonviolent resistance campaigns worldwide and is currently updated to 2019. The introduction of NAVCO has made invaluable contributions to our understanding of popular mobilization. For example, studies utilizing it have revealed a strong positive correlation between nonviolent mass mobilization and subsequent democratization (Chenoweth & Stephan, 2011; Kim & Kroeger, 2019; Rivera & Gleditsch, 2013). Yet, one notable limitation is NAVCO’s exclusive focus on so-called “maximalist campaign goals”, which requires that movements must demand the removal of the sitting regime, the ousting of a foreign occupant, or secession from the state to be recorded. Thus, NAVCO does not systematically document campaigns with more reformist agendas, which, despite falling short of maximalist claims, may still leave significant marks on a regime’s institutional set-up, and, in some cases, even catalyze regime change down the road. Other datasets, such as the *Mass Episodes of Contention* (MEC) data (Chenoweth & Ulfelder, 2017) and the *Nonviolent Episodes and Violent Episodes of Resistance* (NEVER) (Chin, 2017), are similarly constructed.^9^

Another notable campaign-oriented dataset is the *Revolutionary Episodes Dataset* by Beissinger (2022a). It documents characteristics of 345 revolutionary episodes between 1900 and 2014, including the social groups that participated and the goals of the movement. For inclusion, civilian mobilization must amount to a “mass siege” of its government, intending to displace the incumbent regime and substantially alter the political or social order. A *siege* implies the imposition of forceful change, and mere protest mobilization does not suffice. The revolutionary episodes covered are maximalist campaigns and exclude movements with reformist agendas.^10^

Finally, the *Enhanced Major Episodes of Contention* (EMEC) dataset (Kang, 2023) encompasses a broader spectrum of campaigns targeting the government, including reformist initiatives addressing various issues such as food prices, wage disputes, and election laws. Hence, EMEC contains 1613 reformist campaigns and 488 maximalist campaigns, but its time series is limited to 1955–2018.

### The Contributions of OMG

Existing datasets fall short in cataloging, *within the same framework and for different types of movements*, the broader range of movement characteristics highlighted by the more historically oriented scholarship. Key factors include the *ideology* of movements, their *organizational structure*, and their *social composition*; that is, the social and class backgrounds of participants (e.g., industrial workers, students, professionals, public sector employees, peasants). By combining information on these, and other, movement characteristics, OMG will enable researchers to address different interlinkages and potential interactions between them in shaping outcomes such as democratization, state building, or particular institutional reforms. Moreover, OMG will enable researchers to investigate when and under what conditions mass movements take specific forms, involve particular social groups, and adopt distinct ideologies. These questions are relevant for studying historical developments, and one of OMG’s core contributions is extending the potential time period that researchers can study by providing data on mass mobilization movements back to 1789.

OMG even addresses other gaps. First, it goes beyond the exclusive focus on maximalist campaigns that is typical for prior datasets. OMG (also) includes campaigns with less ambitious demands that do not fundamentally challenge the political or territorial integrity of the state but advocate for substantial governance reforms. These include movements seeking greater territorial self-governance or institutional reforms related to civil rights protections, freedom of expression, electoral integrity, or checks on executive authority.^11^ This expanded scope provides significant advantages, capturing campaigns that, while expressing more modest goals, sometimes spark broader and more transformative changes.

Second, OMG categorizes campaign demands based on the movement’s own articulation, assigning them to predefined categories (regime removal, civil rights protection, electoral reform, etc.) without imposing subjective judgments about campaigns’ maximalist or non-maximalist nature more broadly. Determining whether campaigns meet the threshold for maximalist demands has historically proven challenging, and subject to scholarly debate (e.g., Beissinger, 2022b, p. 439), as not all movements that resulted in regime change (and perhaps even intentionally worked towards it) explicitly articulated such goals. For instance, Poland’s Solidarity movement never explicitly called for the removal of the communist regime. During its two active periods in the 1980s, it issued over twenty demands, primarily focused on workers’ rights and economic reforms, alongside calls for electoral reforms, broader freedom of speech, and civil liberties. However, it never directly demanded the regime’s overthrow.^12^

Third, OMG is not restricted to considering campaigns with demands for “liberalizing” institutional changes but also includes campaigns advocating for *less* inclusive institutions or rights protection, or more autocratic regimes. While the conventional view of nonviolent movements often presumes they aim for more inclusive political systems, history shows that some nonviolent campaigns have mobilized, for instance, to marginalize specific groups. For example, as part of the ethnic conflict in western Myanmar, nationalist Buddhist monks campaigned to denounce the existence of the muslim Rohingya population, mobilizing more than 100,000 people.^13^ Notably, many anti-liberal campaigns arise alongside liberal ones, often as reactions to them. Thus, even when analyzing the wider consequences of liberal mass movements, it is relevant to account for such (reactionary) movements. Lastly, OMG encompasses more than “opposition movements.” From 1900 onwards, it also includes pro-regime campaigns aimed at maintaining the status quo or empowering the incumbent regime.^14^ Assessing the make-up, strategies, and effectiveness of pro-regime movements is another fruitful venue of empirical research opened up by OMG.

## Contents of OMG

OMG covers 151 countries, with the longest time series extending from 1789 to 2019. For a comprehensive list of countries and their specific time series, we refer to Appendix B.1. To identify polities and their relevant time series, we relied on the V-Dem dataset and its detailed definitions of country-units (Coppedge, 2022; Coppedge et al., 2022). The reasons for doing so are pragmatic. First, identifying and consistently delineating country-units, especially historically, is difficult and resource-demanding work, which V-Dem has thoroughly done already. Second, V-Dem will, for many purposes, be an important source of covariates for OMG, and matching up exact country-units in, for example, regression analysis mitigates error and missingness due to listwise deletion.^15^ Altogether, we coded 1452 campaigns consisting of 1809 campaign-phases. The occurrence of multiple phases within a single campaign mainly reflects inactive years between active large-scale mobilization. In the following, we discuss the most important definitions that guide the identification of units, as well as OMG’s core variables. More extensive discussions – both conceptual, operational as well as exemplifications – are provided in the Codebook and Rules-of-Thumb documents.^16^

### Identifying the Units

The key unit of OMG is the campaign, although data is also broken down at the campaign-phase level. We define a campaign as *“one or more related and temporally contiguous events of coordinated mass collective action within a polity unit, aimed at least in part at altering or stabilizing/strengthening the current political regime, removing the head of state or government, or altering the territorial composition of the polity.”* We consider events of mass collective action as relevant for inclusion only if they have identifiable start and end points, along with *some* level of coordination.^17^

Additionally, campaigns must meet specific requirements related to *size* and *demands*. For size, a movement must mobilize more than 1000 individuals across various activities of violent or nonviolent resistance (demonstrations, sit-ins, strikes, etc.) within a single calendar year. If at least one year meets this threshold, any contiguous year with over 500 participants is also considered active.^18^

Concerning demands, we require that a campaign must challenge *central aspects of the existing political system or the polity’s territorial integrity* to qualify for inclusion. Thus, movements advancing claims directed at entities other than the regime or state are excluded, as are those mobilizing solely from within the state apparatus, such as military coups. A key stipulation is that the campaign’s demands must challenge “foundational aspects” of the political system, thereby excluding mere policy demands such as parental leave quotas or specific economic concessions. Finally, the campaign’s target polity must correspond to its location; for example, a movement located in Germany demanding regime change in Ethiopia will not be included, even if it meets both demand and size criteria. There are two notable exceptions to this rule: we include (otherwise qualifying) campaigns in (a) *occupied polities* directed at the regime in the occupying polity and (b) cross-polity demands in interlinked polity unions (e.g., Swedish-Norwegian Union, 1814-1905) or colonizer-colonized polity pairs.

To be considered as challenging the *foundation of the political system*, a movement’s demands must specifically fall within one of the following categories: (i) **substantially increased territorial self-governance**, that is, demands to devolve decision-making from the central state to the regional territory; (ii) **secession**, that is, demands for regional withdrawal from the state and the creation of a new state; (iii) **remove the sitting head of state or head of government** (alone or together with other parts of political leadership, such as the cabinet); (iv) **remove the sitting regime**, where the regime is defined as the set of rules that are essential for selecting political leaders and for maintaining them in power; (v) **institutional demands**, which target changes to (or the preservation of) formal institutions and must align with one of the following five sub-categories: civil rights demands, freedom of expression demands, election related demands, demands related to constraints on executive power, and demands related to the exercise of political power.^19^

Following these rules, and as a unique feature of OMG that we discussed in the prior section, we also include (from 1900) so-called pro-regime campaigns and campaigns pursuing less inclusive institutions – that is, advocating for restrictions on one of the five institutional dimensions included in OMG. These campaigns are readily distinguishable in the dataset, allowing researchers to include or exclude them from their analyses depending on the relevance of these campaigns for their research objectives. The same flexibility applies to, for example, opposition campaigns with specific demands.

### Campaign Characteristics

In total, OMG contains more than 100 variables coded at the campaign-phase level.

Below, we briefly discuss four key variable-clusters related to demands, social composition, tactics, and ideology.

#### Demands

As noted, OMG includes movements that challenge foundational aspects of a political system. Under this heading, we record several more specific demands or stated campaign goals, falling under any of the five major categories already described above: substantially increased territorial self-governance; secession; remove the head of state/government; remove the regime; institutional demands. Importantly, these demands enter not only as defining characteristics for which campaigns are included; OMG also contains several variables with detailed information on specific demands, including distinctions on which demands (within a given campaign phase) we consider primary and secondary ones. Beyond categorizing stated campaign demands with 11 distinct variables, we collect textual information on the more specific nature of the demand and demand-drift within campaign phases. We code demands based on what the movement asks for rather than based on any outcome of the movement. Coding explicit demands is important for several reasons, notably improving reliability and avoiding biases from campaigns being coded retrospectively based on (perceived) campaign outcomes or subsequent correlated developments (e.g., observing national-level democratization after the campaign).

#### Social Composition

OMG provides detailed information on the social composition of campaigns, capturing varying levels of involvement across an extensive list of social groups. These groups include peasants, other rural workers, agrarian elites, industrial workers, non-industrial urban workers, public sector employees, business owners, professionals with occupations requiring specialized higher education, intellectuals, students, regime security forces, and members of religious, ethnic, or other ascriptive identity groups. In so doing, we build on, but also extend and provide several nuances to, the scheme used by Dahlum et al. (2019) for coding the social composition of NAVCO campaigns. For each group, we code whether members *participated* in the OMG-recorded movement at some point, *initiated* the movement, and whether the group *dominated* the movement. For participation, we follow Dahlum et al. (2019) in requiring only that sources explicitly indicate that (members of) the group participated in campaign activities. For campaign domination, we apply a more stringent criterion: members of the relevant social group must either constitute a majority of campaign participants *or* exert a critical influence on the campaign strategies and outcomes, with a high threshold for assessing influence as “critical”.

#### Tactics

Mirroring existing campaign datasets, OMG offers a binary metric to classify campaign tactics as predominantly nonviolent or violent (by campaign phase; see the codebook for details on operational rules, including the categorization of specific tactics). However, this binary classification does not reflect the nuances and complexity of (sets of) campaign tactics that are often employed. To address this, OMG contains several additional variables. To account for supplementary tactics we, for example, include one variable indicating the presence of nonviolent activities during predominantly violent campaigns and another for violent activities during predominantly nonviolent ones. Related variables provide additional information on organizational aspects (e.g., organized violent wing), the prevalence of supplementary tactics (by number of participants), and evidence of weapons acquisition and training.

#### Ideology

The OMG dataset records the ideology of a movement if the movement itself or a credible source claims the movement adheres to an ideology within our pre-defined categorical scheme, or if it is highly plausible – based on our interpretation of sources (including movement statements) – that the campaign holds this ideology.^20^ We display time trends for the different ideology categories below. Since campaigns may hold or mix important elements from several of our ideological categories (e.g., nationalism *and* liberalism), a campaign can be recorded with more than one ideology.

#### Other Movement Characteristics

Beyond the four variable clusters, OMG contains several measures that register other features of mass mobilization movements, such as size and organizational structure. The latter include the presence of discernible coordinated leadership and the involvement of different formal organizations, including women’s, students’, religious, and political organizations.^21^

## Data Collection Process and Data Quality

The data collection process for OMG was funded by four major research grants and extended over three years, engaging six researchers and twelve research assistants (RAs) affiliated with the University of Oslo (UiO) and the Peace Research Institute Oslo (PRIO). The UiO team coded historical data spanning from 1789 to 1900, whereas the PRIO team coded 1900–2019. To reduce missingness, when possible, we assigned countries based on RAs regional expertise and language skills. To ensure within-dataset consistency we facilitated extensive coordination between the RAs, also across the two teams, including weekly or bi-weekly virtual and physical meetings between all coders and selected researchers and a shared Teams channel, actively used by all RAs and researchers. Further facilitating cross-team coordination, the two teams were located in the same city and several RAs participated both in the UiO and PRIO team, at different points in time. Furthermore, two RAs were assigned the task of coordinating and overseeing the coding efforts for each of the two timeframes. These RA coordinators were responsible for supervising the coding sheets and coding justification documents, meticulously reviewing each one. The historical and contemporary RA coordinators also spent considerable time in dialogue, reviewing and adjusting coding heuristics and outcomes to ensure consistency across the coding periods. After resolving any issues identified by the coordinators, sheets and coding justification documents underwent review by one of the researchers. These measures were intended to facilitate inter-coder reliability and over-time consistency (three researchers reviewed coding both for the contemporary and historical periods), but also to enhance validity by highlighting good coding practices and establishing solutions to specific challenges along the way. An extensive training-coding scheme for new RAs (on similar cases) and double-coding of selected cases further contributed to these purposes. Appendix E discusses an assessment of inter-coder reliability, based on the coding of 28 campaigns by multiple RAs. We ran this test after the RAs had considerable coding experience, to reflect the consistency and quality of the coding in the final dataset. The rate of agreement was generally very high (.85 mean accuracy rate between pairs of coders, across variables).

The research team jointly developed a pilot version of the codebook, which was used for trial coding of selected countries. Based on these experiences, we revised the codebook to improve and clarify categories, key terms, and instructions for coding. In addition to the codebook, the OMG documentation includes a detailed Rules-of-Thumb document.^22^ This document was instrumental in guiding coders to apply similar heuristics when dealing with complex cases, especially in instances where the coding rules were too intricate to be fully specified in the codebook. It will also help researchers who want to scrutinize, replicate, or expand on our coding.

**Figure 1.**
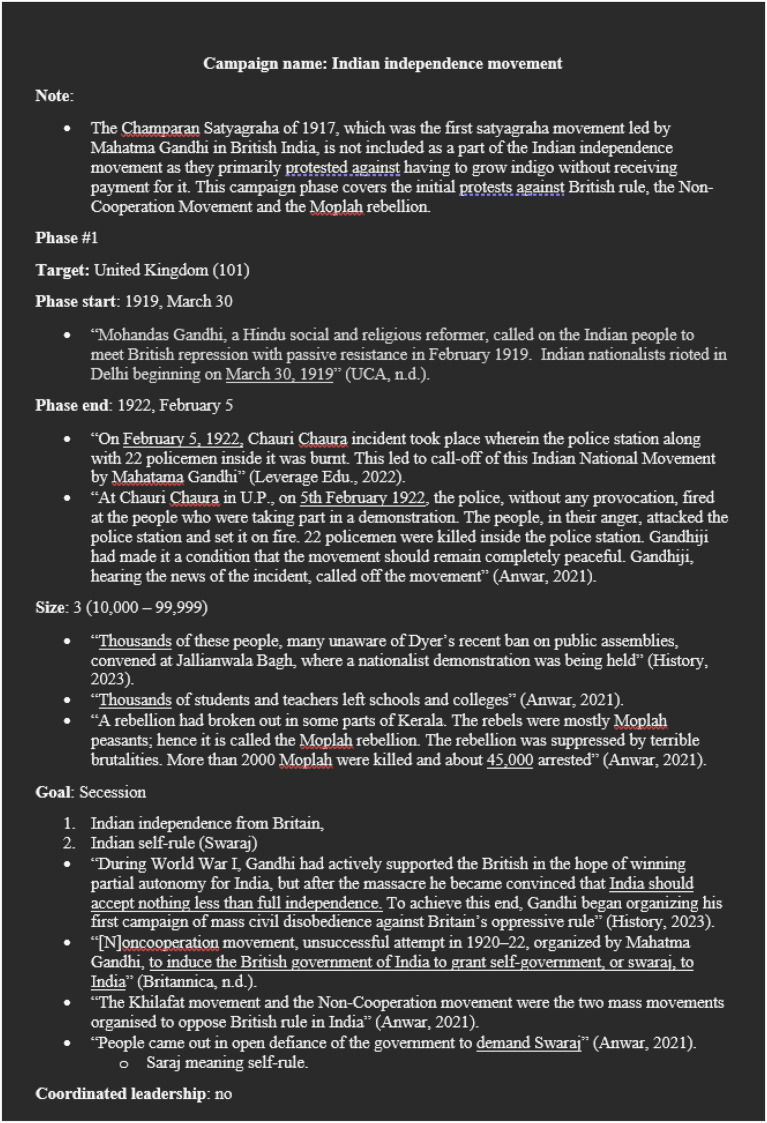
Excerpt from the documentation of the coding of the first phase of the Indian.

Another tool that enhances reliability and validity *and* serves as documentation for outsiders is the comprehensive and similarly structured country-specific coding justification documents. These are sorted first by campaign and then by groups of variables. Figure 1 provides an excerpt from the India document, elaborating on the coding of the Independence Movement's first phase.

These documents, amounting to approximately 4500 pages (2.2 million words), describe, justify, and provide sources for all coding decisions, thereby enhancing transparency. They also constitute rich narrative reports of a country’s social movement history. Their standardized structure and comprehensive content could be helpful for users, beyond validating our data; they could aid in the construction of new variables (e.g., by using AI tools) and allow for text analyses. Concerning subjective interpretations of the sources and reliability, OMG also records how certain the coder was in their coding of some of the main variables. In these measures, we differentiate between lack of information and difficulty in interpreting available information. We also register primary coder IDs for each campaign unit.

To identify potential campaigns, RAs were provided with instructions to consult several sources – many pre-specified – including existing datasets, encyclopedias such as Wikipedia and Britannica, news search engines, research articles, and books. They then assessed which candidate campaigns met the inclusion criteria, before using country-specific sources to gather more detailed information about these campaigns. The total number of unique references registered by the RAs is 8735. Despite our best efforts, the identified sources did not always allow us to code all campaign characteristics. Certain variables, such as social group participation, were harder to code than others, leading to substantial missingness (Appendix Figures B.3 and B.4 provide missingness maps).^23^ For other variables, such as ideology, there is no decisive way to distinguish between missing information about ideology versus a movement not having any ideology (corresponding to our ideology categories). Oftentimes, both instances have the same observable implication in the available source material, namely no clear mentioning of an ideology. Missingness is non-random: in general, sources seem easier to identify for larger, more recent, and more violent movements, and for movements in larger countries. Such movements are thus easier to code in a comprehensive manner.

This latter issue also pertains to *identifying* relevant movements.^24^ While we have tried to be as thorough as possible, certain movements – especially in earlier decades and in smaller countries with non-European languages – are likely not described in the relevant source material at all, or such source material exists, but we have been unable to identify it. Sometimes descriptions of potentially relevant movements exist, but the sources are insufficiently detailed about core components of our rules, such as movement demand or size, for identifying units. This presumably means that we must leave several relevant movements out of the dataset, but we include them in the accompanying “candidate list”.^25^ Users should be aware of these patterns and either try to model the missingness or qualify their conclusions accordingly.

A full overview of country coverage, descriptive statistics for all variables, and missingess patterns, is available in Appendix B.

## Mass Mobilization Across Modern History

In this section, we showcase the OMG data by describing various global historical trends. Despite pertaining to important phenomena and developments, these trends have not been fully detailed, and sometimes not even recognized, by previous studies. This is because the underlying data material has had a more limited temporal scope or because we consider features of mass mobilization movements not covered by other datasets. First, we examine the relative frequency of (predominantly) violent and nonviolent movements back to 1789. Second, we use OMG’s new variables to display and discuss how the typical ideologies of mass opposition campaigns have shifted through modern history. Third, we map the dominant social groups in mass mobilization movements across time. Fourth, we describe trends in movements’ organizational structures, including the involvement of civil society organizations and political parties, as well as the presence or absence of a unified, coordinated leadership.

### Independence Movement

**Figure 2.**
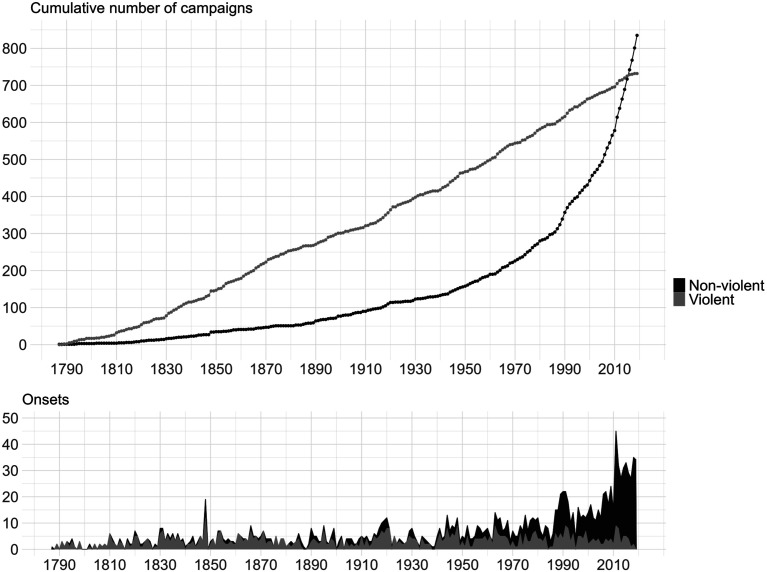
Opposition campaigns with violent versus nonviolent tactics. The upper panels shows the cumulative number of onsets, while the lower panel shows the yearly number of onsets. Movements which main demand pertains to supporting the current regime or government are not included.

We consider, first, campaign tactics, and how they have evolved historically. Figure 2 shows the cumulative number of mass *opposition* movements, divided by whether they are predominantly pursuing violent or nonviolent tactics, as defined by OMG. Given our focus on opposition movements in this exercise, all movements whose main demand pertains to *supporting* the regime or government are removed from these counts. Appendix Figure B.5 shows a more detailed picture by plotting time trends also for predominantly violent campaigns with a nonviolent flank (e.g., a guerrilla movement with a political party) and predominantly nonviolent campaigns with a violent flank. Here, we focus on the global time trends in main strategy.

Figure 2 tells a fairly straightforward story about the evolution of mass opposition campaigns, globally.^26^ The late 18th and most of the 19th century were primarily marked by violent opposition campaigns: in the 1890s, the cumulative number of registered violent campaigns was about five-fold that of nonviolent ones. After 1945, and especially after 1960, the number of new nonviolent opposition campaigns globally has dramatically outpaced the number of violent ones. In 1960, the cumulative numbers of violent and nonviolent opposition campaigns recorded in OMG were 499 and 190, respectively. These numbers turned identical in 2016, and in 2019 they reached 732 and 835, respectively.

The trend towards more nonviolent opposition campaigns in recent decades is known from before (e.g., Chenoweth, 2020), but the addition of a longer historical time series highlights the gradual and drawn-out nature of this development and puts the recent, substantial increase in an even more dramatic perspective. The increased nonviolent mass mobilization aligns with Meyer and Tarrow’s (1998) description of the emerging *movement society*, where social protest has evolved to become a more prevalent and routine feature of political bargaining in advanced democracies, over time mobilizing more diverse constituencies around more diverse claims. It indicates a structural shift in the nature of political contention, taking place over a span of 200 years of human history.

One interpretation of the observed trends from Figure 2 is the following: As organizational knowledge and technological innovations evolved, and as more specific knowledge about how to effectively organize nonviolent mass movements spread across the world, nonviolent mobilization became a much more popular way to engender substantial political change, especially from the 1980s onwards (for a nuanced discussion on the time-varying effectiveness of nonviolent campaigns, see Chenoweth, 2020).

Yet, there is another (methodological) interpretation of the trend, which we believe is likely a complementary (rather than a strictly alternative) explanation of the recent, rapid increase in observed nonviolent opposition campaigns. The proliferation of digital and other media sources in recent years has facilitated the detection of contemporary campaigns. This, coupled with plausible assumptions about which types of campaigns are relatively easier and harder to detect, indicates that a “registration bias” might contribute to the observed pattern in Figure 2. Without going into detail, we surmise that violent and large-scale events tend to be more noticeable and, thus, better documented in archival material or by academics in articles and history books compared to their nonviolent and smaller counterparts. Advancements in information technology and the expansion of media outlets may, however, have enhanced the visibility of less dramatic events in recent years. Therefore, the apparent increase in nonviolent campaigns depicted in Figure 2 might, in part, result from the historical under-reporting of smaller, nonviolent activities, which are now more readily documented due to the sheer amount of easily searchable source materials. In short, violence leaves a bigger trace for posterity to spot, but technological developments have evened out the resulting differential between violent and nonviolent activities. Such registration biases may exist despite the extensive efforts and resources used to code historical cases, and such bias would likely influence any type of data collection on this phenomenon (expert coding, text mining, etc.). We will return to the potential consequences of such a bias and how to assess it in our application of nonviolent versus violent campaigns and subsequent democratization.

**Figure 3.**
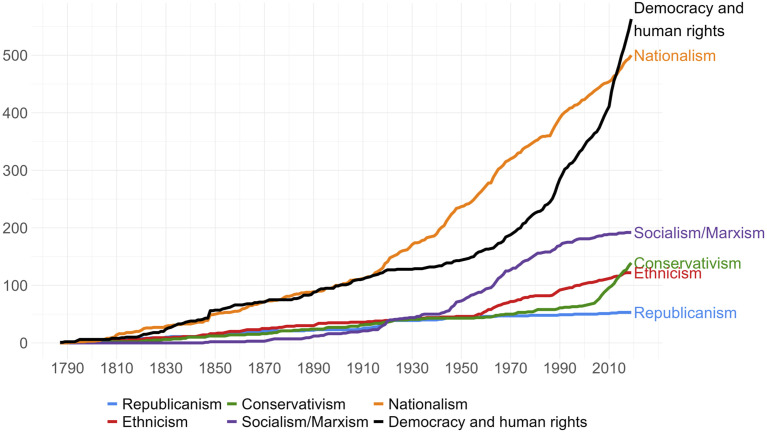
Cumulative number of campaigns over time, by ideology.

**Figure 4.**
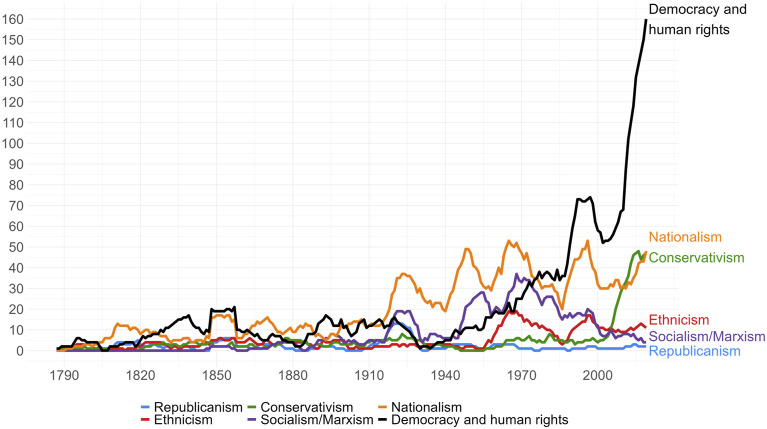
Number of campaigns over time, by ideology, sum of the past 10 years.

Second, we turn to OMG’s six-category ideology measure to explore changes in the (typical) ideological outlook of campaigns over time. Figure 3 displays the cumulative number of campaigns, globally, with particular ideologies from 1789 to 2019. Complementing this picture, Figure 4 presents 10-year moving sums of campaigns categorized by ideology. Appendix Figure B.6 illustrates trends in the *shares* of campaigns with different ideologies, while Appendix Figures B.7 and B.8 show these trends for opposition movements only. We remind that our categorization scheme allows campaigns to be coded with multiple ideologies.

Briefly summarized, throughout the 19th century the ideologies of campaigns featured a balanced mix of nationalism, conservatism, and republicanism/liberalism, particularly in Europe and Latin America. From the early 20th century, socialism emerged as the predominant ideology, alongside nationalism, before its prominence began to decline from the 1970s. In more recent decades, ideologies centered around human rights and democracy have taken the forefront, even surpassing other ideologies in terms of cumulative count. Moreover, nationalism has consistently been a prevalent ideology throughout the observed time period and also across different regions. Consequently, the cumulative count of nationalist campaigns is thus still quite close to the cumulative number of democracy and human rights campaigns.

**Figure 5.**
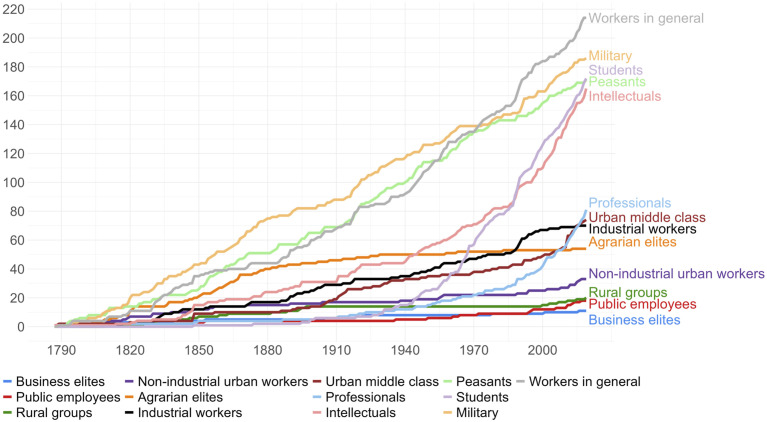
Cumulative number of campaigns over time, categorized by dominating social group.

Third, we consider trends in dominant social groups in mass mobilization movements. Figure 5 shows the cumulative number of campaigns, globally, with different dominating groups – from our 13-category scheme – across 1789–2019. A similar illustration restricted to opposition movements only is presented in Appendix Figure B.9. Figure 5 highlights the broad diversity among dominating social groups, revealing that, as of 2019, no single group has dominated in more than $\frac{1}{8}$ of campaigns. This variation can be observed throughout history, with, for example, urban workers, military actors, and peasants all being frequently recorded as the dominating group since the early 19th century.

Especially since the 1980s, however, students and intellectuals have increasingly become the dominating social groups in mass opposition movements. In fact, the number of observed campaigns with these groups dominating in the forty years after 1980 is comparable to the 190 preceding years.^27^ Conversely, other groups have diminished in relevance over time. The most notable example is agrarian elites, who were among the most frequently recorded dominating groups before 1890, but have only dominated a handful of mass opposition movements since then.

Fourth, we consider the organizational characteristics of typical mass mobilization movements and how they have evolved. Organizational capacities are widely believed to profoundly impact movement dynamics and success rates (Chenoweth et al., 2017; Pinckney et al., 2022; Sutton et al., 2014), as well as the possibility of lasting democratic change (Kadivar, 2018). Interestingly, the recent decline in the “success rate” of nonviolent opposition movements has partly been attributed to a shortage of organization and leadership, with a growing reliance on social media for coordination (Chenoweth, 2020). Although several scholars have suggested a weakening of the organizational infrastructure of nonviolent movements (Tufekci, 2017), extensive data that could be used to substantiate (or contest) this claim have been limited. The OMG data allows for a more systematic evaluation of trends in the organizational structure of movements.

**Figure 6.**
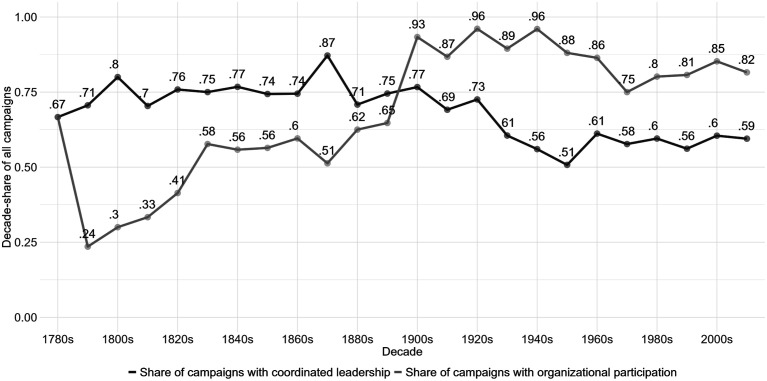
Share of all campaigns by decade with participation from a civil society organization or party (light grey), and share of all campaigns by decade with coordinated leadership (dark grey).

In Figure 6, we assess trends in two organizational characteristics of campaigns:^28^ the share of campaigns including at least one civil society organization or party and the share of campaigns with coordinated leadership. The latter pertains to whether there exists a person or smaller collective body of authority that coordinates the majority of the campaign’s participants and key activities. Figure 6 reveals that these two organizational characteristics have followed very different trajectories. The proportion of campaigns involving at least one civil society organization or party has increased from the start of the 19th century (30% of campaigns) to the present (80%), but has been consistently high going back to 1900.^29^ In contrast, the share of organizations with centralized leadership remained fairly flat, around 75%, percent during the 19th century, but has thereafter declined. Yet, the decline was gradual, occurring primarily from the 1920s to the 1950s. Since the 1960s, the share of such campaigns has remained stable at around 60% – a trend that continued into the 2010s. Thus, the vast majority of campaigns from 1789 to the present have featured centralized leadership. Even in today’s digital era, with its extensive opportunities for decentralized coordination through social media and other communication technologies, leadership and hierarchical structures continue to characterize the majority of mass mobilization movements.

## Nonviolent Mobilization and Democracy

Finally, we use OMG data to revisit and refine our understanding of the relationship between nonviolent mobilization and subsequent democratization, illustrating how juxtaposing information on different campaign features pertaining to social composition, aims, ideologies, and tactics can improve our understanding of political development. In brief, the link between nonviolent mobilization and democratization is robust, also when using our data. Yet, we find that this relationship is conditional on the types of social actors participating in mass movements as well as their stated demands and ideology.

Perhaps the most influential scholarly work within the civil resistance literature, Chenoweth and Stephan (2011), reported that nonviolent strategies outperform violent ones in achieving their objectives, which often include democratization. This finding has been highly influential in both academic- and policy circles (see, e.g., Merriman et al., 2023) and subsequent work has expanded on this result (Dahl & Gleditsch, 2023; Kim & Kroeger, 2019; Pinckney, 2021; Rivera & Gleditsch, 2013). While some of this research points to caveats and qualifications, the prevalent notion is that nonviolent campaigns are more likely to bring about political change and, in particular, more democratic forms of government.

Yet, almost all large-N studies of nonviolent action and democratization rely exclusively on one dataset, NAVCO. Recent research has questioned the robustness of the *correlations* between nonviolent movements and “movement success” or democratization (see, e.g., Anisin, 2020; Lehoucq, 2016; Dworschak, 2023), suggesting they may be sensitive to the inclusion of a few unrecorded campaigns (Dworschak, 2023) and, more generally, to unit selection, coding idiosyncrasies (such as threshold for registering violence), or measurement errors. These concerns highlight the need for additional robustness tests on datasets with distinct inclusion criteria, operational rules, and coding practices.

While the OMG data do not provide a panacea for addressing these various threats to inference, its long time series, careful methodology, and inclusion of additional campaigns and campaign characteristics enable us to mitigate – or at least assess – several concerns. The extended temporal coverage allows us to explore whether nonviolent campaigns contributed to democratization also before 1900 or if this pattern is circumscribed to the 20th and 21st centuries. Moreover, the relationship between nonviolent mobilization and democratization is usually studied under strong assumptions of homogeneity, with limited attention paid to the diverse identities, ideologies, or demands of movements engaging in nonviolent mobilization. Some research suggests that the success of certain movement tactics is conditional on protesters’ identities (e.g., Manekin & Mitts, 2022), but this notion remains to be thoroughly explored in observational data on mass movements. Using OMG data, we examine the heterogeneity of effects of nonviolent mobilization on democracy, considering movements’ social composition, claims, and ideologies.

Our main independent variables register the mobilization of *predominantly* violent- or nonviolent opposition movements.^30^ These variables are recorded as two dummy variables at the country-year level, where 1-scores denote the presence of *one or more* campaigns employing the respective (predominant) strategies. Focusing on opposition movements, we omit campaigns mobilizing in support of the current regime or government. Our outcome variable is V-Dem’s Polyarchy index (Teorell et al., 2019), which measures electoral democracy on a 0–1 scale and is constructed from five sub-indices measuring, respectively, the extents to which key political offices (e.g., legislators) are elected, elections are free and fair, suffrage is provided, freedom of speech and alternative information are ensured, and freedom of association is protected.^31^ In all models, we control for (lagged) Ln population and Ln GDP per capita (from Fariss et al., 2022) as well as pre-treatment Polyarchy scores. Furthermore, we control for the simultaneous presence of other predominantly violent and nonviolent movements, oppositional or not.

To address causal inference challenges, we adopt a panel-matching difference-in- differences approach, following the PanelMatch framework developed by (Imai et al., 2021). This method identifies a suitable control unit for each treated observation at time *t*, using cases from the *same time period* with *similar treatment histories* and with *similar pretrends*. We then divide the dataset into sub-groups based on countries’ treatment status (the onset or not of violent/nonviolent campaigns) for the 10 years prior to treatment. Following the exact matching procedure, we ensure that each subset is balanced using propensity-score matching on the covariates (Ln GDP per capita, Ln population, presence of other movements of different types) and the pre-treatment outcome (Polyarchy) over the same 10-year period (Imai & Ratkovic, 2013). Subsequently, we create synthetic counterfactual outcomes for each treated unit (i.e., units with nonviolent campaigns when considering the effects of such campaigns) using the weighted averages of the matched control units. We then calculate the difference-in-difference estimate for each treated unit before averaging these estimates across all treated units. The resulting measure is known as the Average Treatment Effect on the Treated (ATT). To examine both the short- and medium-term effects of nonviolent and violent campaigns, we estimate the ATT for 0–10 years after the first year a campaign-phase begins. While this is not a foolproof technique for causal inference – it relies on non-trivial assumptions, as all other relevant techniques and designs – it represents an improvement on previous attempts to draw causal conclusions about the impact of nonviolence on democratization, while allowing us to diagnose the validity of its underlying assumptions.

**Figure 7.**
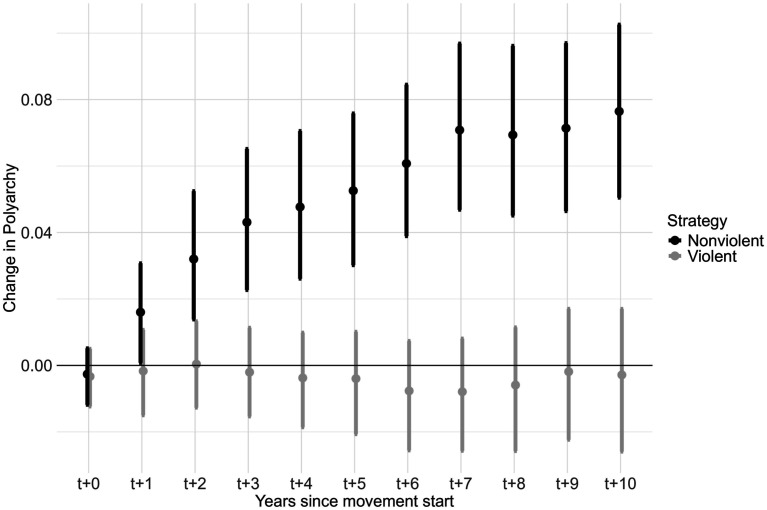
ATT for violent versus nonviolent protest opposition movements on subsequent Polyarchy-scores. Points indicate the point-estimate for the ATT of violent (grey) and nonviolent (black) movements on Polyarchy (Y-axis) in 0–10 year (X-axis) after the first year of the movement. The bars represent the 95% confidence interval for the pointestimate.

Figure 7 plots our benchmark results for nonviolent and violent opposition movements. Analyzing data from 199 nonviolent opposition movement onsets where we can construct an appropriate comparison case, we find that – on average – countries experiencing a nonviolent movement subsequently became relatively more democratic. This relationship is statistically significant at conventional levels from the first year after the campaignphase started, and the size of the estimated effect and t-value increase further over time. After 10 years, the Polyarchy score of these countries is estimated to have improved by around 0.075 on average – around 30% of the index’s standard deviation – compared to the counterfactual scenario with no such movement. Conversely, violent mobilization, of which our procedure identifies 197 situations with a suitable comparison, shows no association with subsequent changes in democracy: the ATT is close to 0 and statistically insignificant for all years following the campaign. Additional information about these results, including tabular presentations, is available in Appendix C.1.

Granted, violent and nonviolent mobilization might systematically co-vary with other forms of instability, as might democratization. To account for potential confounding, we thus rerun the benchmark models controlling also for independent statehood, extent of territorial control, ongoing civil war, and the number of previously successful military coups. Our main results remain unchanged when accounting for these instability measures (see Appendix C.2).

Yet, the pre-treatment trends for both violent and nonviolent opposition movements indicate that onsets are more likely to occur in more autocratic countries that experience a deterioration in democratic institutions. Appendix Figure C.1 illustrates the covariates’ pre-trends for both models.^32^ Note that these patterns violate the so-called parallel trends assumption, indicating that we should be careful with drawing causal inferences. Yet, the level of imbalance is relatively small, with the largest standardized weighted mean difference being −0.06.

Another cause for concern is the potential influence of reporting biases on the observed relationship. The plausible pattern that “successful” nonviolent campaigns are registered more frequently than unsuccessful ones could skew any analysis considering the impact of nonviolent campaigns. However, modern information technology has likely made it easier to detect events and developments, including smaller nonviolent movements with minimal political impact. Therefore, if observation bias is the primary driver of the observed relationship between nonviolent campaigns and democratic change, one might expect the positive ATT of nonviolent movements to be more pronounced in historical periods where detection is (presumably) harder. There may, of course, still be a reporting bias today, but we surmise that the bias should be larger further back in time.

**Figure 8.**
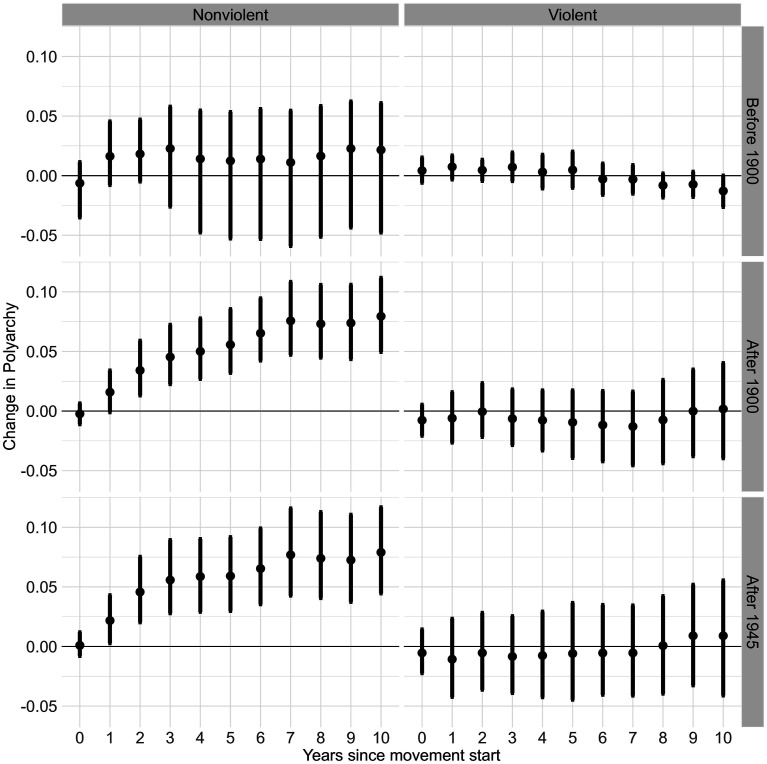
ATT for violent versus nonviolent protest movements on subsequent Polyarchyscores for different historical time periods. Points indicate the point-estimate for the ATT of the respective combination of strategy and era on Polyarchy (Y-axis) in 0–10 years (X axis) after the first year of the movement. The bars represent the 95% confidence interval for the point-estimate.

Figure 8 presents separately estimated ATTs for violent versus nonviolent campaigns for three time periods: 1789–1899, 1900–2019, and post-World War II. Extensive historical reporting bias would predict a larger effect for nonviolent movements in the early period. This is not the case. There is no clear association between nonviolent opposition movements and democratization before 1900. In the 20th and 21st centuries, there is. This divergence across samples *could* be due to some other type of reporting bias, which is stronger for the 19th century, but we find this less plausible. Another interpretation is that the effectiveness of nonviolent mobilization has varied over time, for instance being more effective in the 20th than in the 19th century, potentially due to conditional factors such as the prevalence of particular communication and organization technologies, but we leave this question for future research. Nevertheless, our results suggest that the link between nonviolence and democratization does not reach back into the 19th century.

The impact of nonviolent movements may also depend on specific movement characteristics. OMG offers unprecedented opportunities to assess such potential heterogeneity, for instance pertaining to the stated demands of movements. Not all movements aim to change their country’s institutions, and some may want to do so, but refrain from voicing such demands for strategic reasons. Although improvements in democracy may occasionally be a “by-product” of nonviolent movements with other demands, we anticipate that democratic improvements will primarily result from movements with explicitly stated demands to liberalize regime institutions. The OMG dataset delineates five demand categories: territorial autonomy, secession, removing the sitting government, removing the current regime, or institutional changes.

**Figure 9.**
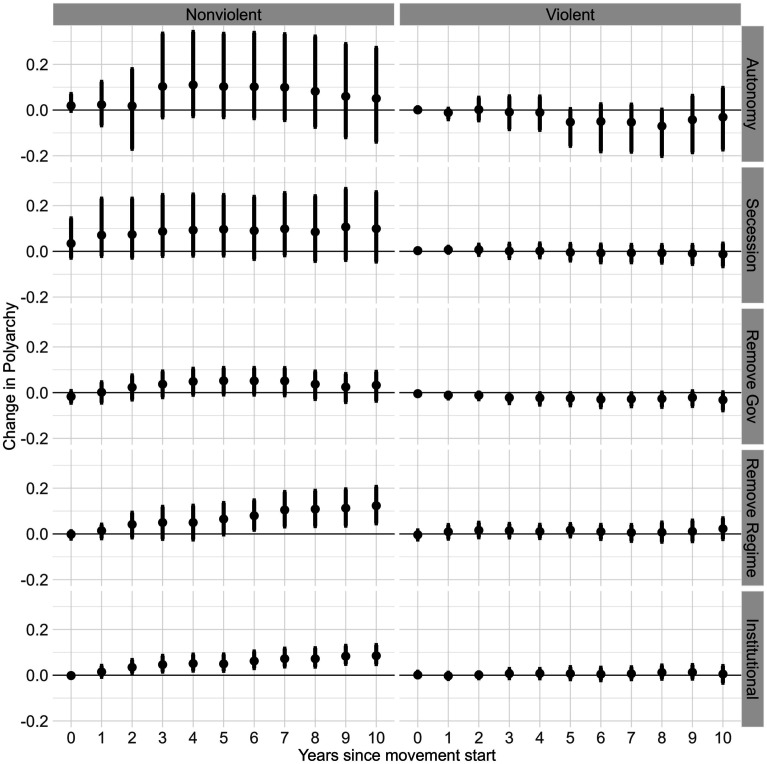
ATT for violent versus nonviolent opposition movements on subsequent Polyarchyscores, grouped by the demand of the movement. Points indicate the point-estimate for the ATT of the respective combination of strategy and demand on Polyarchy (Y-axis) in 0–10 year (X-axis) after the first year of the movement. The bars represent the 95% confidence interval for the point-estimate.

Following our benchmark setup, we estimate 10 different models, each with the treatment being a combination of violence/nonviolence and one of the five main demands. The results, presented in Figure 9, reveal statistically significant ATTs for nonviolent movements with regime removal or institutional change as main demands. Notably, the effects of nonviolent movements with institutional-change demands are on par with those of regime-change demands. This underscores the need to incorporate non-maximalist demands into datasets mapping mobilization. Omitting them risks overlooking critical pathways through which grassroots movements reshape political systems and contribute to democratization. Conversely, nonviolent movements mainly demanding increased autonomy, secession, or government removal are not significantly related to subsequent democratization. Similarly, none of the five categories of violent mass mobilization demonstrate a significant association with democratization.

**Figure 10.**
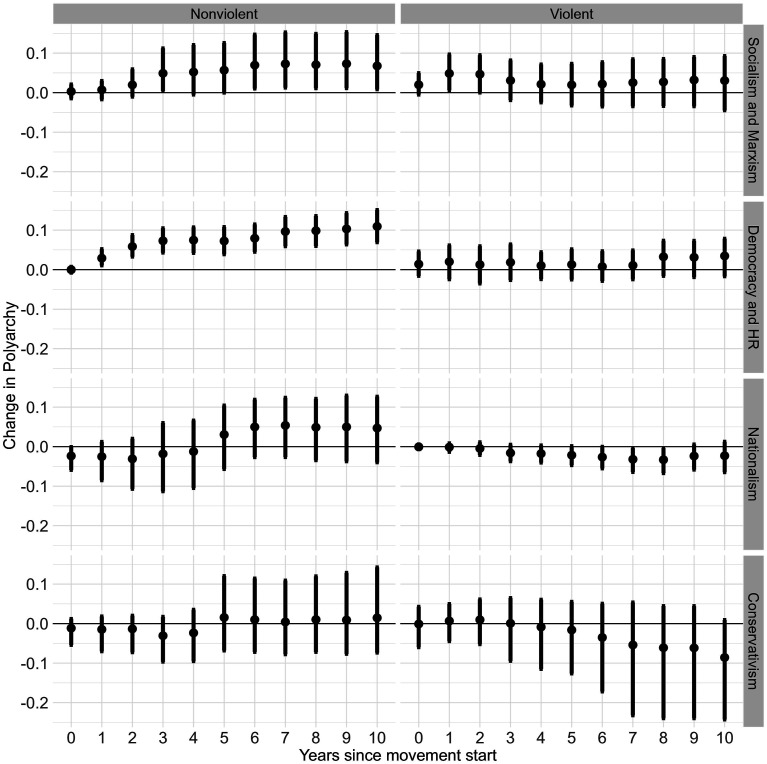
ATT for violent versus nonviolent protest movements on subsequent Polyarchyscores, grouped by ideology. Points indicate the point-estimate for the ATT of the respective combination of strategy and ideology on Polyarchy (Y-axis) in 0–10 year (X-axis) after the first year of the movement. The bars represent the 95% confidence interval for the point-estimate.

Next, we assess whether nonviolent campaigns with particular ideologies more likely engender democratization. This is certainly plausible, insofar as ideologies entail normative ideas about hierarchies of power, with implications for different institutions relevant to democratic rule. We once again follow our benchmark setup, but now differentiate between onsets of (violent or nonviolent) campaigns with four ideologies: socialism/marxism, democracy and human rights, nationalism, and conservativism. The results in Figure 10 suggest that the positive relationship between nonviolent mobilization and subsequent democratization depends on the mobilizing group’s ideology. Countries that experience nonviolent campaigns with socialist or marxist ideologies or, especially, democracy and human rights ideologies, are more likely to see subsequent democratization. Nonviolent nationalist or conservative campaigns are unrelated to democratization. Violent mobilization does not systematically correlate with democratic improvements for any ideology. Indeed, violent nationalist campaigns are seemingly detrimental for democracy.

**Figure 11.**
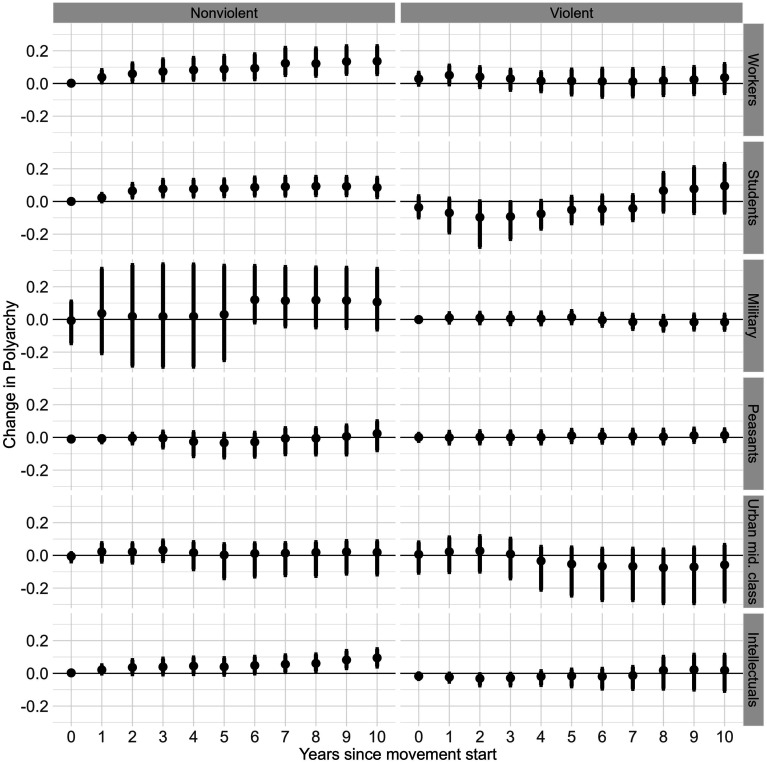
ATT for violent versus nonviolent protest movements on subsequent Polyarchyscores, grouped by the dominating social group. Points indicate the point-estimate for the ATT of the respective combination of strategy and social group on Polyarchy (Y-axis) in 0–10 year (X-axis) after the first year of the movement. The bars represent the 95% confidence interval for the point-estimate.

Beyond demands and ideology, does the mobilization of particular social groups moderate the relationship between nonviolent mobilization and democratization? Following our benchmark setup, we estimate the ATT on democracy for the onset of subsets of violent and nonviolent campaigns by whether campaigns were dominated by urban workers, students, military personnel, peasants, urban middle classes, or intellectuals. The results in Figure 11 indicate that violent mobilization has no discernible relationship with democratization regardless of dominating group. In contrast, democratization is expected after nonviolent campaigns dominated by workers, student or intellectuals, but not by military personnel, the urban middle classes or peasants.

To sum up, we find support for the relationship between nonviolent mobilization and democratization when using the new OMG data. Yet, the specification including all types of nonviolent movements fails to satisfy the parallel trends assumption, casting some doubt on whether this estimate represents a causal effect of nonviolent mobilization on democratization. Through further inquiry, we delineate several scope conditions to the associations between nonviolent movements and democratization, pertaining to campaign demands, ideology, and the dominating social group. In these more nuanced analyses, we find clear evidence that certain types of nonviolent movements enhance democratization, but not others. Importantly, we do not find evidence suggesting that any particular subset of violent movements is related to democratization. This latter finding may, to some extent, reduce concerns that previous findings reflect that nonviolent campaign strategies are merely spuriously associated with democratization due to some favorable selection process. Readers should keep in mind that across several sub-group analyses, the number of treated countries is much lower than in the aggregated analysis: 9 out of the 36 sub-analyses presented have 15 or fewer treated countries after matching (see appendix C). The lack of more (matched) treated countries underscores the relative infrequency of some of these movements and, consequently, how uncertain we should be about their associations with democracy.

## Conclusion

We have presented the Opposition Movements and Groups (OMG) dataset, covering 1452 mass mobilization movements from 151 countries between 1789 and 2019. More specifically, we described the dataset’s contents and how it fills important gaps left by existing datasets. OMG facilitates a unique data portrait of mass movements throughout the modern era. Utilizing various OMG measures, we highlighted notable historical patterns of change as well as persistence in movement ideology, social group participation, and organizational characteristics globally over the past 230 years. We also used OMG data to re-assess the relationships between nonviolent and violent movements and subsequent democratization, concluding that certain types of – albeit not all – nonviolent movements enhance the likelihood of democratization.

Let us take one step back and elaborate on the potential significance of collecting extensive data on mass movements across modern history. Democracy datasets such as Polity and V-Dem extend back in time to cover the 19th century (and further back to 1789 for V-Dem). These datasets have opened up for the systematic study of regime institutions, also before the 20th century, and have thus contributed substantially to our knowledge about the development, causes, and effects of democracy. Similar cross-country time-series data on key political actors operating outside established institutions, including mass movements, have been scarcer, which has made it harder to obtain systematic and empirically founded knowledge about, for instance, opposition actors compared to institutions or actors occupying formal political offices.

This data lacuna, we believe, does not reflect the lack of importance of mass mobilization movements and their characteristics for explaining political development. For instance, the 1789 French Revolution and 1917 Russian Revolution demonstrate how mass movements can lead to the overthrow of political regimes and the establishment of new ones. These movements and events may also shape political and social life, more broadly, for years to come. Mass movements sometimes act as critical junctures (see, e.g., Pierson, 2000) that shake up social and political orders and put countries on new development paths. As the French- and Russian revolutions are testaments to, mass opposition movements can thus shape how institutions look and work several decades, even centuries, afterwards.

We contend, instead, that the lack of data on specific features of opposition movements reflects the complexity and resource-intensive nature of classifying and coding these characteristics reliably and validly across historical and geographical contexts. Thus, historical mass opposition movements, especially before the NAVCO dataset (and still today for pre-1900 movements), have typically been studied through qualitative comparative and case studies (notable examples are Skocpol, 1979; Collier, 1999b; Rueschemeyer et al., 1992). These works provide in-depth insights into mass mobilization dynamics and the influence of particular movements and groups in shaping the politics of individual countries. Yet, such studies alone will not enable us to answer descriptive questions about global patterns or to conduct systematic analysis of the antecedent or subsequent correlates of opposition movement characteristics. This is why the invention of NAVCO (Chenoweth & Stephan, 2011) was so important for knowledge development, spurring a cottage industry of empirical studies.

Yet, even the comprehensive NAVCO dataset “only” goes back to 1900 and thus leaves out large parts of modern history. If we, for example, are to understand how and why the established democracies of many current OECD countries came about, which arguably has ramifications for how they look and function today, we need to reach further back in history. Hence, one aim underlying the collection of OMG was allowing for more comprehensive historical descriptions and analysis of mass mobilization movements throughout modern history. Moreover, we anticipate that different variables in OMG will open up for the systematic study and more thorough assessment of several research questions of great importance. Let us mention four examples:

First, the social group coding in OMG may enable researchers to assess which particular constellations of classes and interests are effective in spurring democratization, national independence, or other types of major change, once they coordinate and mobilize. Second, the ideology coding in OMG could be used to study, for instance, whether movements with particular ideologies, such as liberalism, conservatism, or socialism, tend to precede particular types of regimes or (regimes that pursue) particular types of policies. Third, by using data on movement demands, researchers could assess whether the existence of movements with particular demands (e.g., on particular civil liberties) enhances the chances of subsequent movements with similar or even more expansive (e.g., regime change) demands down the line. Fourth, since existing datasets have mainly covered campaigns with “maximalist” demands related to regime change and autonomy, it remains an open empirical question whether previously identified conditions spurring campaign onsets or campaign success are similar for campaigns with less extensive claims, such as limited suffrage expansion or modifications in the electoral system.

These are only a few examples of intriguing research questions that can be addressed with our new dataset. The relevance of understanding mass mobilization seems as potent as ever, even if the focus is restricted to current developments. Quite recently, authoritarian regimes in Iran (the Women Life Freedom movement) and China (the Hong Kong protests) were rocked by mass mobilization. And, as the Black Lives Matter protests or the student protests against the war in Gaza illustrate, the phenomenon is also highly relevant in democracies such as the United States. Understanding the history of mass mobilization, aided by the systematic analysis of extensive data, is crucial to understanding the present social and political situation in countries across the world.

## Supplemental Material

Supplemental Material - Mass Mobilization in the Modern Era: Introducing the Opposition Movements and Groups (OMG) Dataset, 1789–2019 Supplemental Material for Mass Mobilization in the Modern Era: Introducing the Opposition Movements and Groups (OMG) Dataset, 1789–2019 by Marianne Dahl, Sirianne Dahlum, Hanne Fjelde, Haakon Gjerløw, Carl Henrik Knutsen, Carina Strøm-Sedgwick, and Tore Wig in Comparative Political Studies

## Data Availability

Replication data and materials for this article, including the dataset, analysis script, accompanying README file, codebook, narrative reports, and “Rules of Thumb”, are available in the Harvard Dataverse at https://doi.org/10.7910/DVN/G4FCZO (Dahl et al., 2025), with different versions and documentation also available at https://www.prio.org/data/39.

## Notes

1. We use “campaign” and “mass mobilization movement” interchangeably as synonymous terms. While the majority of movements coded in OMG are, indeed, *opposition* movements, the dataset is expanded with several units coded as pro-regime or pro-government movements after 1900. Dataset users should note this compositional change of units in 1900, and account for it, e.g., by filtering out proregime movements when constructing or interpreting plots with historical trends or running regression analysis over the entire time series.

2. Examples of such movements coded in OMG are the French Revolutions of 1789, 1830, and 1848, the Taiping Rebellion of the 1850s and 60s in China, the Bolshevik revolution in Russia 1917, the Indian independence movement, and the anti-Apartheid movement in South Africa.

3. Pro-regime movements are only covered post-1900, and then only if they meet the general criteria for inclusion in the dataset detailed below.

4. OMG distinguishes between a mass movement and the social groups and organizations that underpin them. In this scheme, social groups and organizations refer to (one or more) distinguishable collectives within a movement such as industrial workers (social group) or labor union (organization).

5. Only one dataset, NAVCO 3.0, includes both event-based data and campaign-oriented structures.

6. These pioneering data included country-year level count variables of domestic disturbances such as riots, strikes, and anti-government demonstrations back to 1815. Its extensive coverage is a notable advantage, but CNTS has been criticized for lack of transparency and different validity issues, including geographical biases related to using a single US newspaper outlet (Bethke & Pinckney, 2019; Herkenrath & Knoll, 2011; Rivera & Gleditsch, 2013).

7. Specifically, the *Mass Mobilization dataset* gauges events where 50 or more protesters publicly demonstrate against the government, and records protesters’ demands and state responses (Clark & Regan, 2016). This global dataset extends across 1990–2003. The *Armed Conflict Location & Event Data Project* (ACLED), another global data source, includes violent events, demonstrations, and select (politically relevant) nonviolent events, with time series from 1997 onwards (Raleigh et al., 2010). More violent forms of collective action are included in the *Geo-referenced Event Dataset* (GED) from UCDP, but time-series coverage is limited to 1989 onwards (Sundberg & Mellander, 2013).

8. For instance, the *Mass Mobilization in Autocracies Database* (MMAD) maps sub-national mass mobilization events in autocracies worldwide across 2003–2018 (Weidmann & Rød, 2019). Some data sources cover specific democratic countries, including the *European Protest and Coercion Data* (Francisco, 1996), the *PolDem-Protest Dataset* (e.g. Kriesi et al., 2020), the *Dynamics of Collective Action in the United States* (Earl et al., 2003), and the *Crowd Counting Consortium*. The Social Conflict in Africa Database (SCAD; Salehyan et al., 2012) covers large-scale riots and protests in addition to violent events, but covers only Africa and Latin America. Lastly, the *Urban Social Disorder* (USD; Thomson et al., 2022) dataset records various nonviolent and violent events in 186 national capitals and major urban centers across 1960–2014.

9. NEVER reconstructs and extends NAVCO 2.0, featuring only maximalist campaigns, but including nearly three times as many nonviolent episodes as NAVCO. Similarly, MEC includes only maximalist campaigns, and is available globally from 1955 to 2013.

10. See Appendix D for a more direct comparison of the units covered in OMG, on the one hand, and NAVCO 2.1 and Revolutionary Episodes, on the other. Here, we discuss differences in identified campaigns due to differences in various operational rules, but also differences that are not attributable to such differences but rather to measurement error (in one of the datasets). The former differences are more important, but examples of the latter are several campaigns included by NAVCO (indicating, e.g., they have *>* 1000 participants) and excluded by OMG since we could not find clear evidence in the sources of more than 1000 people participating.

11. OMG excludes campaigns narrowly focused on policy reforms or wage demands, which is covered by the above-discussed EMEC dataset extending back to 1955.

12. Similarly, participants in Georgia’s 2003 Rose Revolution, though ostensibly protesting regime removal in response to a widely perceived unfair parliamentary election, facilitated democratic change by specifically demanding the resignation of President Eduard Shevardnadze and his government.

13. See, for example, https://southeastasiaglobe.com/buddhist-protest-aid-agencies-myanmar-rakhine/.

14. Pro-regime campaigns before 1900 have not been coded due to funding constraints.

15. Due to budget constraints, our coding leaves out about 50 typically smaller polities covered by VDem, including several pre-unification Italian and German polities that V-Dem codes during the 19th century. We hope to cover more polities in future iterations of the dataset, should we receive additional funding.

16. See Appendix A for access.

17. Contrary to NAVCO, we do not restrict our focus to campaigns with a clearly discernible leadership. Instead, we code the presence of unified, discernible leadership, be it a person, group of people, or organization, in a separate variable. This allows for distinguishing more hierarchical and centralized campaigns from “flat-structure” campaigns. OMG also differs from NAVCO in departing from the criterion that events need to extend over more than one day. If all criteria are fulfilled during one day, we include the campaign in OMG.

18. The size requirement, which aligns with several existing datasets, serves a pragmatic purpose: making our data collection feasible. It also enhances data reliability, as detecting large movements is less affected by reporting bias. However, it has substantive implications; for example, violent campaigns might, on average, mobilize fewer active participants than nonviolent ones, potentially making our dataset less representative of the wider universe of movements. We use the term “mass mobilization” to clearly signal the scope condition of our data collection pertaining to the size criterion.

19. The distinction between demands for policy changes versus for civil rights protection or institutional change is not always straightforward. For instance, abortion-related campaigns represent such ambiguous cases, which we have classified as civil rights campaigns, specifically within the sub-category of sexual and reproductive freedoms. However, these can be easily identified and excluded by users of the dataset if desired. Other examples are more clear-cut. The civil rights movement in the US, for example, clearly falls within our requirements. The African American Civil Rights Campaign is divided into five phases, from the Silent March in 1917 to the Black Lives Matter protests in 2014. Across the five phases, the campaign’s main demands were always institutional, relating to electoral or civil rights demands. In contrast, anti-nuclear protests during the Cold War are not included since they pertained to policy demands. However, the anti-nuclear protests in 1960 (and, e.g., the anti-Vietnam war protests in 1965) exemplify entries included in our *candidate list*: a list of all movements that were considered, but ultimately not included, accompanied by a description of why.

20. Justifications of the ideology codings are provided in the detailed case notes, allowing dataset users who disagree with our interpretations to re-code cases.

21. While some existing datasets (e.g. NAVCO) code “movement success”, we deliberately opted not to do so. First, defining movement success is inherently complex, and often pertains to extra-movement entities. For instance, success may relate to broader country-level outcomes, such as democratization. This requires a separate coding scheme, for instance to determine when regime-change occurs, and lies outside of the purview of our data collection. Moreover, there is a more fundamental question of causal attribution inherent in coding “success”; while democratization may follow mass mobilization, disentangling the role played by the movement from other factors is hard to do for RAs and risks hardwiring unwarranted causal assumptions into the coding. To assess “movement success” we surmise that it is better to rely on other datasets crafted for measuring the entities and properties (e.g., democratization) that success pertains to, and explicitly linking these data to OMG data in empirical analysis (as we do in the final application of this paper).

22. Both documents are available at https://osf.io/n38by/?viewonly=857360cdeeee40a0a919bcb9a549bcfa.

23. However, when we considered it justifiable to assign values despite somewhat unclear sources, coders included a detailed explanation for their decision in the country-specific documents and sheet and scored the corresponding *uncertainty* variable as high.

24. An additional, relevant feature is the country’s language matching with our coding team’s (extensive) language competencies. Several early-1900s campaigns in African and Middle Eastern colonies were only identified because coders happened upon them in (sometimes rather obscure) French- or Spanish-language resources. We often struggled to find any indication of these events, even in subsequent targeted searches in English-language sources. The same holds true, e.g., for some Scandinavian pre-1900 campaigns.

25. This list encompasses movements that were under evaluation for inclusion in OMG, but that didn’t meet a specific defining criterion. Should evidence emerge in the future – for example from knowledgeable users of the dataset – that candidates were mistakenly excluded, we will include and code them in later iterations of OMG.

26. Strictly speaking, the units are campaign phases, but we use “campaigns” as short-hand in the discussion.

27. Yet, we remind that the number of countries expands over time, especially in 1900, and that we also code pro-regime movements after 1900.

28. Appendix Figure B.10 illustrates a similar trend with opposition movements only.

29. The jump around 1900 may, in large part, stem from changed sample composition. Many additional polities – especially African and Asian former colonies – are coded only from this date in OMG as well as in V-Dem, which we use for identifying and delimiting time series.

30. OMG also records the presence of violent flanks in predominantly nonviolent campaigns, and vice versa, which could be used for relaxing the threshold for registering violent or nonviolent movements. We conduct additional tests with flanks in Appendix C.2, with results closely aligning with our benchmark results.

31. The majority of indicators entering Poyarchy are expert-coded, typically by five or more V-Dem country-experts, and aggregated to the country-year level through V-Dem’s Bayesian IRT measurement model (for details, see Pemstein et al., 2018). This model produces accompanying uncertainty estimates, driven, e.g., by the extent of expert disagreement. In our setting, measurement error in our dependent variable (Polyarchy) is likely to inflate confidence intervals for our estimated treatment effect, and analysis explicitly incorporating V-Dem’s uncertainty estimates might thus have yielded slightly different results.

32. Comparing the balance for the nonviolent ATT matched panels, treated units exhibit lower Polyarchy scores than untreated ones prior to mobilization. Rather than improving in the years preceding mobilization, Polyarchy scores instead show a slight decline. Such a pre-trend contradicts suspicions of reverse-causality, where non-violent movements would appear in situations where democratic change is already underway.
