# Supplementary material for: Mass Mobilization in the Modern Era: Introducing the Opposition Movements and Groups (OMG) Dataset, 1789–2019
Source: Comp Polit Stud. 2025 Aug 27;59(10):2117–58. doi: 10.1177/00104140251369330 (PMC13427092; doi:10.1177/00104140251369330)
Supplement: Supplemental Material - Mass Mobilization in the Modern Era: Introducing the Opposition Movements and Groups (OMG) Dataset, 1789–2019 [file sj-pdf-1-cps-10.1177_00104140251369330.pdf]

Supporting information

Opposition Movements and Groups 1789-2019: Introducing the OMG Dataset

Table of Contents

A Documentation ii

B Systematized information about the data and additional descriptive analyses ii

B.1 Country coverage . . . ii
B.2 Descriptive statistics . . . ii
B.3 Missing data . . . vii
B.4 Additional trend-graphs . . . ix

C Additional PanelMatch models and pre-trends xiii

C.1 Additional information to models presented in the main article . . . xiii
C.2 Additional PanelMatch analyses . . . xvi

D Comparison between OMG and existing datasets xxiv

E Inter-coder reliability tests xxviii

A Documentation

The following link is to a webpage containing, inter alia, (anonymized versions of) the OMG Codebook, Rules-of-Thumb for coding document, and country-specific files with narrative documentation for all coded campaigns: `https://osf.io/n38by/?view_only=857360cdeeee40a0a919bcb9a549bcfa`

Data Availability Statement
Replication materials will be made available upon publication.

B Systematized information about the data and additional descriptive analyses

B.1 Country coverage

All 151 countries in the OMG dataset were coded according to V-Dem's definition of polity start- and end-dates, with each polity's start-date determined by its first recorded year in V-Dem. Accordingly, 71 were included in the dataset's historical coding, with records beginning in the 18th or 19th century. In addition to outlining the time series for each country, V-Dem's country-coding units document (Coppedge et al., 2022) also delineate the specific areas defined to be within the polity borders at each point in time. We have followed this time-specific definition of countries also when registering the movements in OMG (thereby making our data geographically compatible with V-Dem).

Within the subset of countries included in the historical coding, 1789 is the modal start-year.[34] The remaining 80 countries were coded from various starting points in the 20th century, again adhering to V-Dem's delineation of polity units and start- and end-years. For this group, 1900 is clearly the modal start-year.[35] Figures B.1 and B.2 illustrate the distribution of countries across these two temporal clusters, with black tick-marks indicating the onset of at least one new campaign in the respective year.

B.2 Descriptive statistics

Tables B.1 and B.2 present descriptive statistics for all numerical variables in the OMG dataset. Excluded from these tables are text-based variables, such as the names of participating organizations. We have also excluded all ID variables.

[34] Note that the Abolition movement in the United Kingdom starts in 1787, but is ongoing in 1789, meaning that we code this movement.

[35] Among these countries, Somalia has a campaign starting in 1899, but which is ongoing in 1900, and we thus include it.

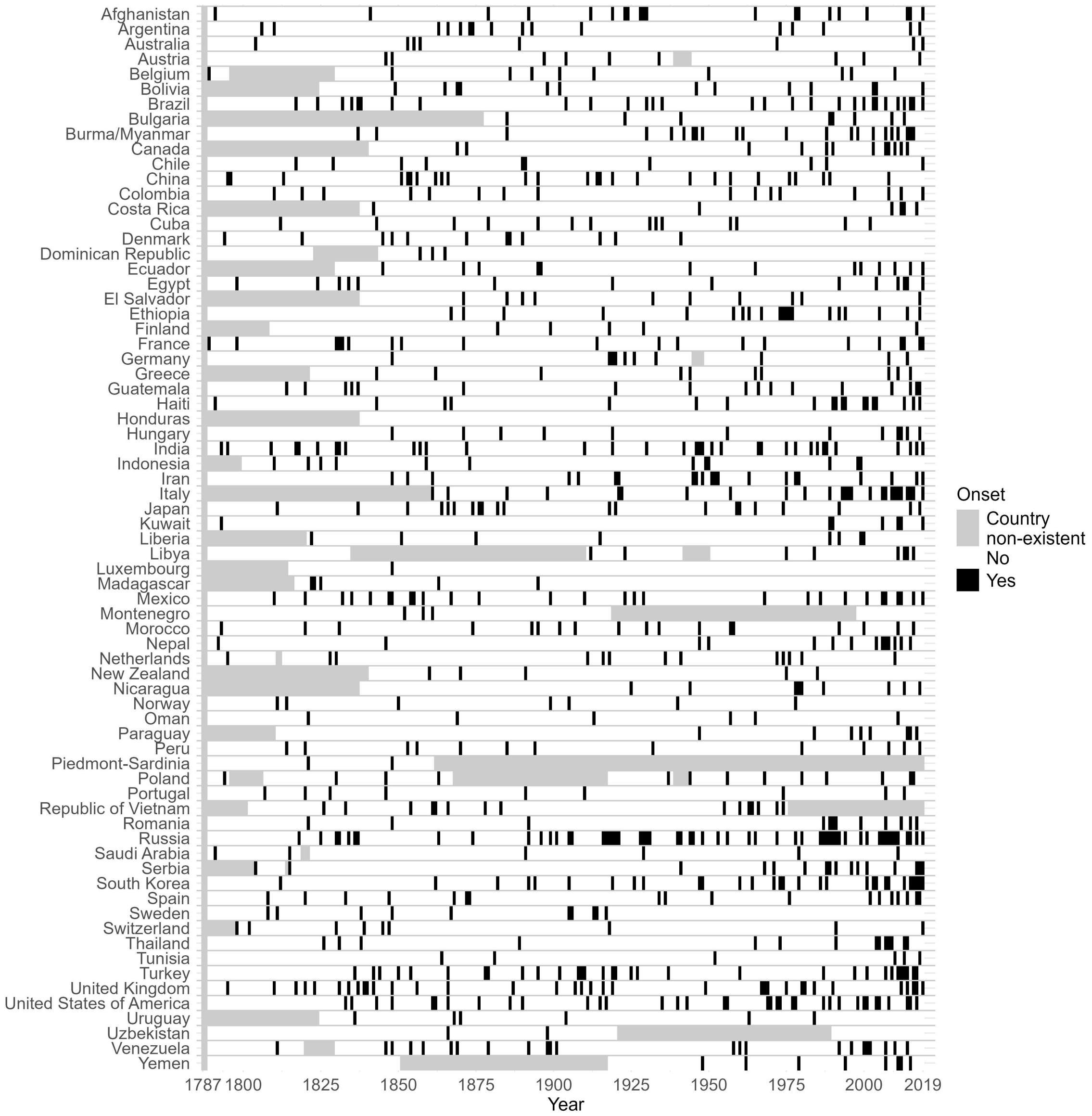

Figure B.1: Campaign onsets in countries coded between 1789-2019

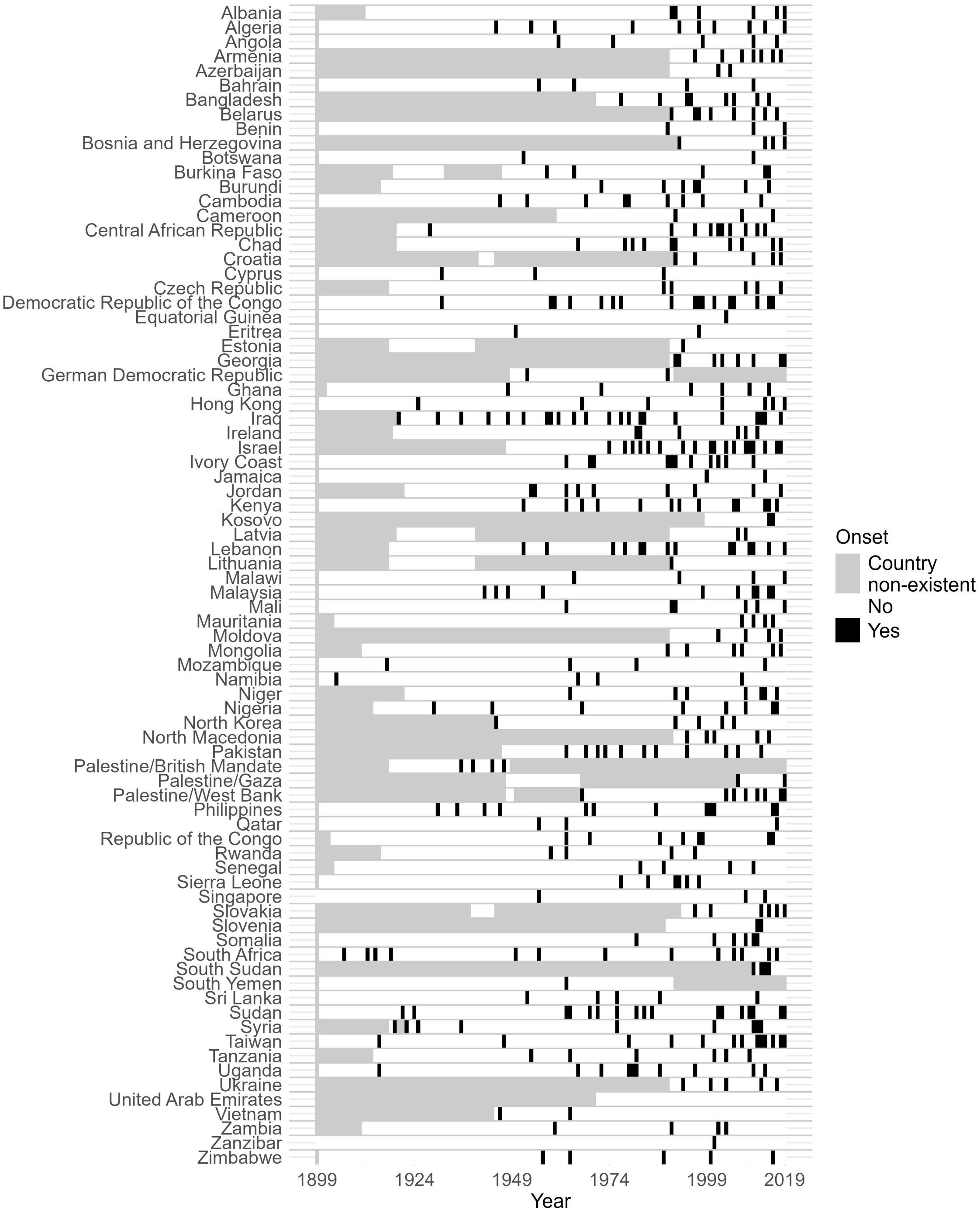

Figure B.2: Campaign onsets in countries coded between 1900-2019

Table B.1: Descriptive statistics for numeric variables

Group	Variable	Min	1st qty.	Median	3rd qty.	Mean	Max	SD
Demand	Democracy	0	0	0	0	0.2	1	0.4
	Autonomy	0	0	0	0	0.08	1	0.27
	Secession	0	0	0	0	0.17	1	0.37
	Regime change	0	0	0	1	0.33	1	0.47
	Anti-regime change	0	0	0	0	0.05	1	0.22
	Institutional: Political power	0	0	0	0	0.09	1	0.29
	Institutional: Civil rights	0	0	0	0	0.21	1	0.41
	Institutional: Anti-civil rights	0	0	0	0	0.05	1	0.21
	Institutional: Electoral changes	0	0	0	0	0.19	1	0.39
	Institutional: Anti-Electoral changes	0	0	0	0	0	1	0.05
	Institutional: Constrain executive	0	0	0	0	0.04	1	0.2
	Institutional: Anti-constrain executive	0	0	0	0	0	1	0.06
	Institutional: Freedom of expression	0	0	0	0	0.07	1	0.25
	Institutional: Anti-freedom of expression	0	0	0	0	0	1	0.03
	Remove government	0	0	0	1	0.8	3	1.2
	Anti-remove government	0	0	0	0	0.12	1	0.32
	Remove government: any	0	0	0	1	0.36	1	0.48
	Remove government: cabinet	0	0	0	0	0.21	1	0.41
	Remove government: HOS only	0	0	0	0	0.13	1	0.34
	Remove government: part of cabinet	0	0	0	0	0.02	1	0.15
	Main: autonomy	0	0	0	0	0.04	1	0.2
	Main: institutional	0	0	0	0	0.19	1	0.39
	Main: Anti-institutional	0	0	0	0	0.04	1	0.2
	Main: remove government	0	0	0	0	0.22	1	0.41
	Main: Anti-remove government	0	0	0	0	0.09	1	0.29
	Main: remove regime	0	0	0	0	0.23	1	0.42
	Main: Anti-remove regime	0	0	0	0	0.03	1	0.18
	Main: secession	0	0	0	0	0.15	1	0.35
Ideology	Conservative	0	0	0	0	0.08	1	0.27
	Democracy and HR	0	0	0	1	0.31	1	0.46
	Ethnicity	0	0	0	0	0.07	1	0.25
	Nationalist	0	0	0	1	0.28	1	0.45
	Republican	0	0	0	0	0.03	1	0.17
	Socialist marxist	0	0	0	0	0.11	1	0.31
	Pride-antipride campaign	0	0	0	0	0.02	1	0.14
Organizational participation	Civil society, other	0	0	0	0	0.17	1	0.37
	Labor union	0	0	0	0	0.16	1	0.37
	Regime security forces	0	0	0	0	0.02	1	0.15
	Military veterans	0	0	0	0	0.01	1	0.12
	Other	0	0	0	0	0.21	1	0.41
	Political party	0	0	0	1	0.35	1	0.48
	Rebels	0	0	0	1	0.29	1	0.46
	Religious	0	0	0	0	0.11	1	0.31
	Student	0	0	0	0	0.1	1	0.29
	Women	0	0	0	0	0.08	1	0.28
Other	Coordinated leadership	0	0	1	1	0.63	1	0.48
	Peak participant size	1	2	3	3	3.01	6	1.03
	Size without petition	0	2	3	3	2.98	7	1.07
Religion	Other	0	0	0	0	0.02	1	0.15
	Buddhist	0	0	0	0	0.01	1	0.08
	Catholic	0	0	0	0	0.03	1	0.18
	Islamist	0	0	0	0	0.07	1	0.25
	Protestant	0	0	0	0	0.01	1	0.11
Time	End month	1	4	7	11	7.23	12	3.62
	End precision	1	1	1	1	1.39	4	0.82
	End year	1790	1918	1983	2009	1957.59	2019	62.46
	Start date	1	3	12	21	12.91	31	9.47
	Start month	1	3	5	9	5.64	12	3.5
	Start precision	1	1	1	1	1.21	3	0.53
	Start year	1787	1917	1978	2007	1955.11	2019	62.22

Table B.2: Descriptive statistics for numeric variables continued - Social group and strategy variables

Group	Variable	Min	1st qty.	Median	3rd qty.	Mean	Max	SD
Social group dominate	Agrarian elites	0	0	0	0	0.03	1	0.18
	Business elites	0	0	0	0	0.01	1	0.08
	Industrial workers	0	0	0	0	0.04	1	0.21
	Intellectuals	0	0	0	0	0.1	1	0.31
	Military employees	0	0	0	0	0.12	1	0.32
	Non-industrial urban workers	0	0	0	0	0.02	1	0.14
	Peasant	0	0	0	0	0.11	1	0.31
	Professionals	0	0	0	0	0.05	1	0.22
	Public employees	0	0	0	0	0.01	1	0.11
	Religious or ethnic	0	0	0	1	0.38	1	0.48
	Rural workers	0	0	0	0	0.01	1	0.11
	Students	0	0	0	0	0.11	1	0.31
	Urban middle class	0	0	0	0	0.05	1	0.21
	Workers general	0	0	0	0	0.14	1	0.34
Social group originate	Agrarian elites	0	0	0	0	0.06	1	0.23
	Business elites	0	0	0	0	0.01	1	0.12
	Industrial workers	0	0	0	0	0.04	1	0.19
	Intellectuals	0	0	0	0	0.17	1	0.38
	Military employees	0	0	0	0	0.14	1	0.35
	Non-industrial urban workers	0	0	0	0	0.02	1	0.14
	Peasant	0	0	0	0	0.07	1	0.25
	Professionals	0	0	0	0	0.09	1	0.28
	Public employees	0	0	0	0	0.02	1	0.14
	Religious or ethnic	0	0	0	1	0.34	1	0.47
	Rural workers	0	0	0	0	0.01	1	0.09
	Students	0	0	0	0	0.11	1	0.31
	Urban middle class	0	0	0	0	0.04	1	0.19
	Workers general	0	0	0	0	0.12	1	0.32
Social group participation	Agrarian elites	0	0	0	0	0.11	1	0.32
	Business elites	0	0	0	0	0.16	1	0.37
	Industrial workers	0	0	0	0	0.15	1	0.35
	Intellectuals	0	0	0	1	0.44	1	0.5
	Military employees	0	0	0	1	0.32	1	0.47
	Non-industrial urban workers	0	0	0	0	0.24	1	0.43
	Peasant	0	0	0	1	0.28	1	0.45
	Professionals	0	0	0	1	0.39	1	0.49
	Public employees	0	0	0	0	0.14	1	0.35
	Religious or ethnic	0	0	1	1	0.54	1	0.5
	Rural workers	0	0	0	0	0.06	1	0.24
	Students	0	0	0	1	0.39	1	0.49
	Urban middle class	0	0	0	0	0.14	1	0.35
	Workers general	0	0	0	1	0.41	1	0.49
Violence strategy	Violent activity	0	0	0	1	0.41	1	0.49
	Violent activity 1000	0	0	0	0	0.08	1	0.28
	Violent activity 25	0	0	0	0	0.22	1	0.41
	Violent flank	0	0	0	0	0.08	1	0.27
	Weapon acquisition	0	0	0	0	0.07	1	0.25
	Weapon training	0	0	0	0	0.01	1	0.11
	Nonviolent activity	0	0	0	1	0.28	1	0.45
	Nonviolent activity 1000	0	0	0	0	0.19	1	0.39
	Nonviolent activity 25	0	0	0	0	0.22	1	0.42
	Nonviolent flank	0	0	0	0	0.17	1	0.37

B.3 Missing data

Figures B.3 and B.3 display missing data patterns for all variables. ID variables and time-related variables (start- and end-dates) are excluded as they have no missing information by definition. Note that violent-flank variables are only coded for primarily nonviolent campaigns, and vice-versa. For all observations, one group of flank-variables are therefore missing by definition. Similarly, names for political parties, rebel groups, and other organizations are missing by definition if no such groups participated in the campaigns.

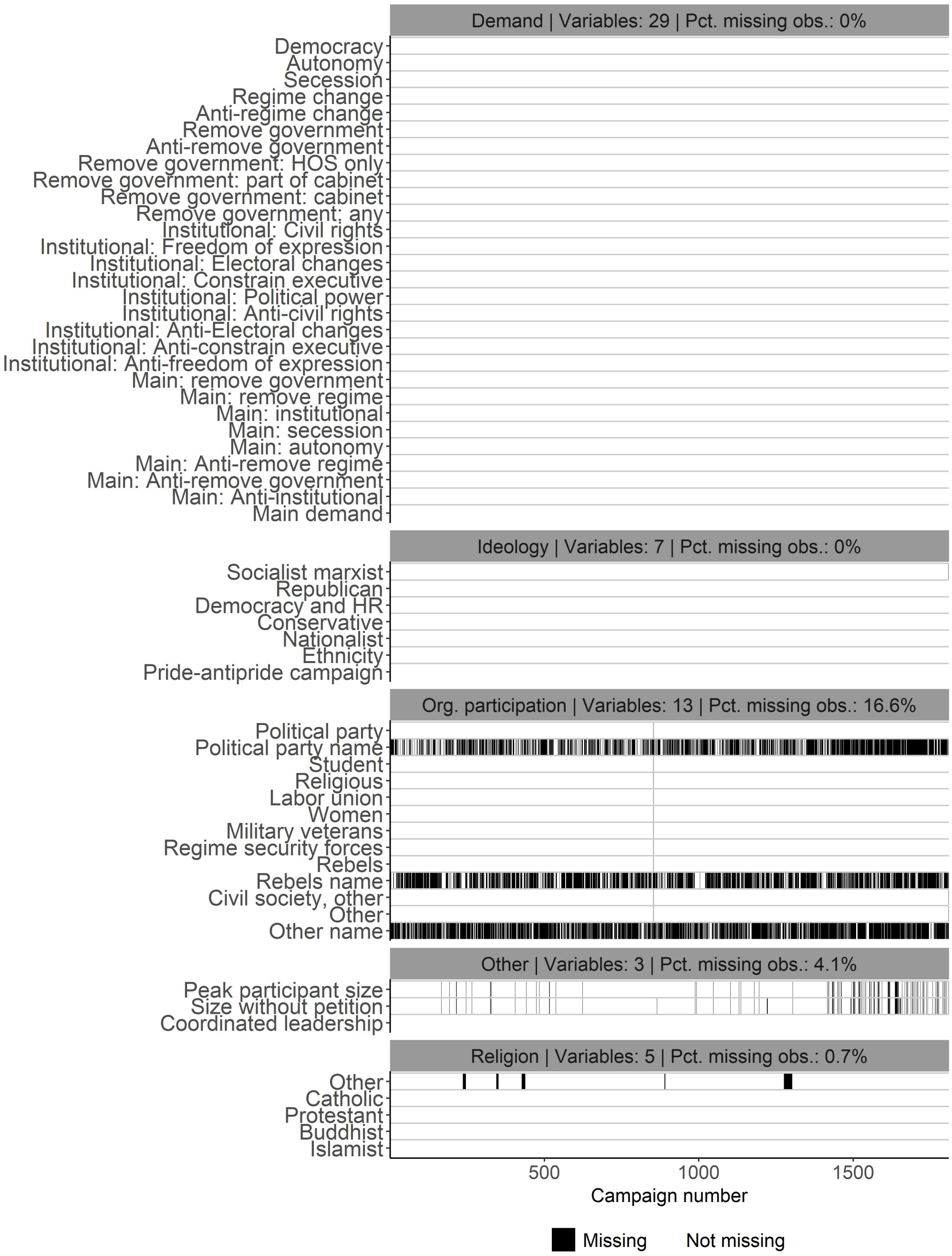

Figure B.3: Missing values across groups of variables

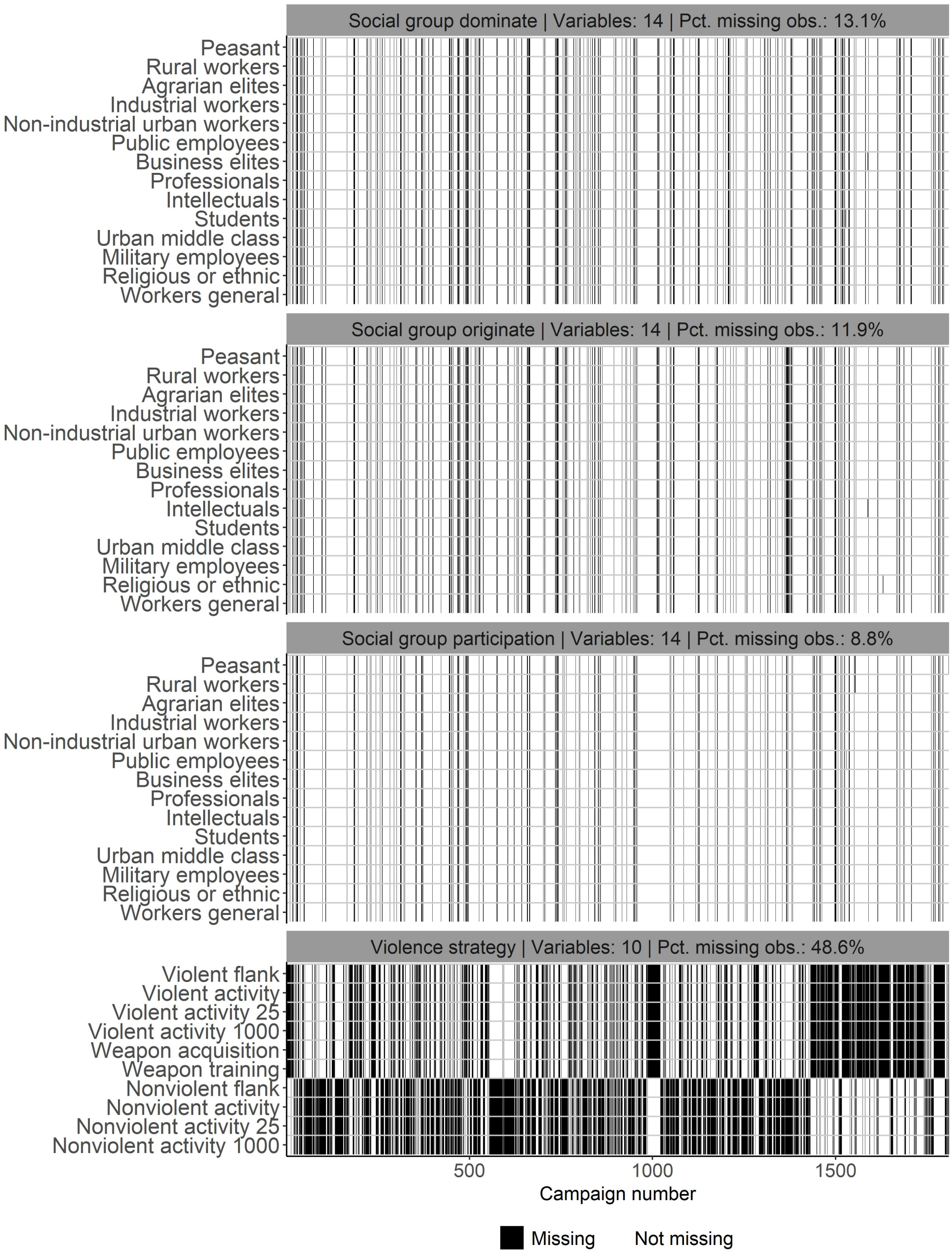

Figure B.4: Missing values across groups of variables, continued: Social group and strategy variables

B.4 Additional trend-graphs

In this section, we present supplementary graphs that are commented on but not included in the main article. Figure B.5 shows the cumulative number of campaigns divided by more complex categorizations of violent versus nonviolent strategy, now including whether the campaign also had flanks employing the opposite strategy to the campaign's dominant strategy (e.g., nonviolent campaign with violent flank).

Figure B.6 illustrates the proportion of campaigns associated with each ideology over the past 10 years. To ensure accurate interpretation, we note again that our ideology coding allows for a campaign having multiple ideologies.

The subsequent figures display similar trends to in the main paper, but focusing only on opposition campaigns instead of also including pro-regime campaigns.

Strategy flanks

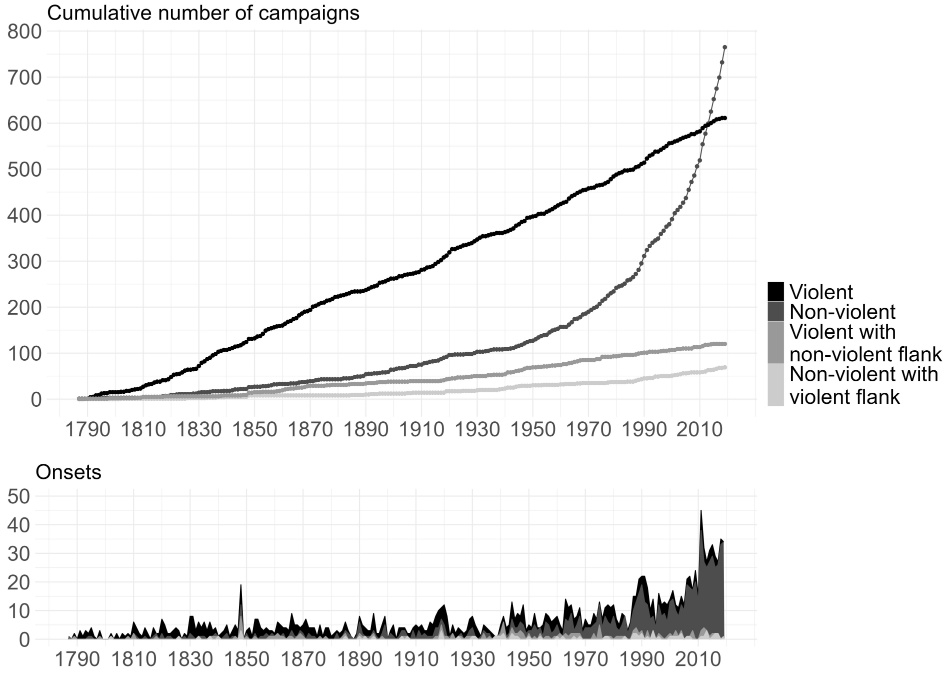

Figure B.5: The upper panel shows the cumulative count of campaigns by year, categorized by their predominant strategy– violent or nonviolent,–and whether they included a flank employing the opposite strategy. The lower panel displays the annual number of onsets for each type.

Campaign ideologies

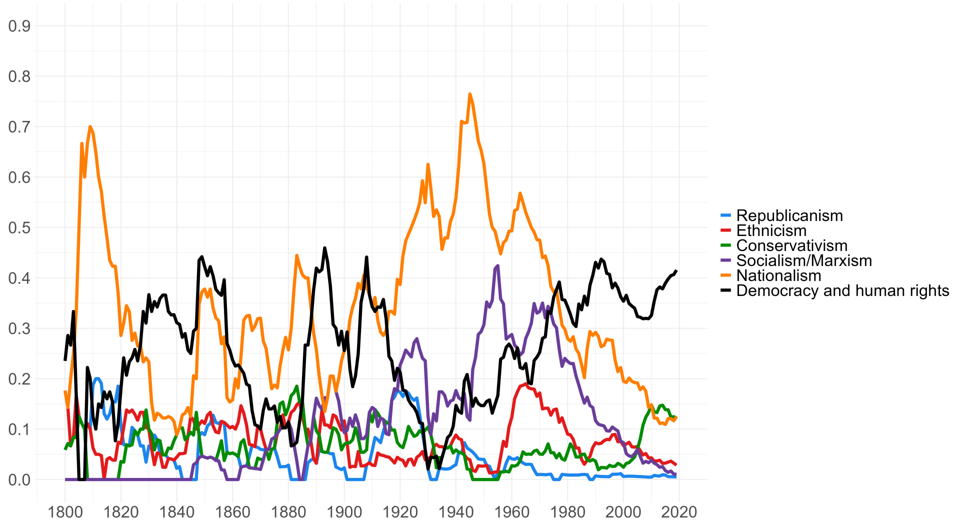

Figure B.6: Campaigns by ideology over time, share of total campaigns over the past 10 years

Cumulative ideology, opposition movements only

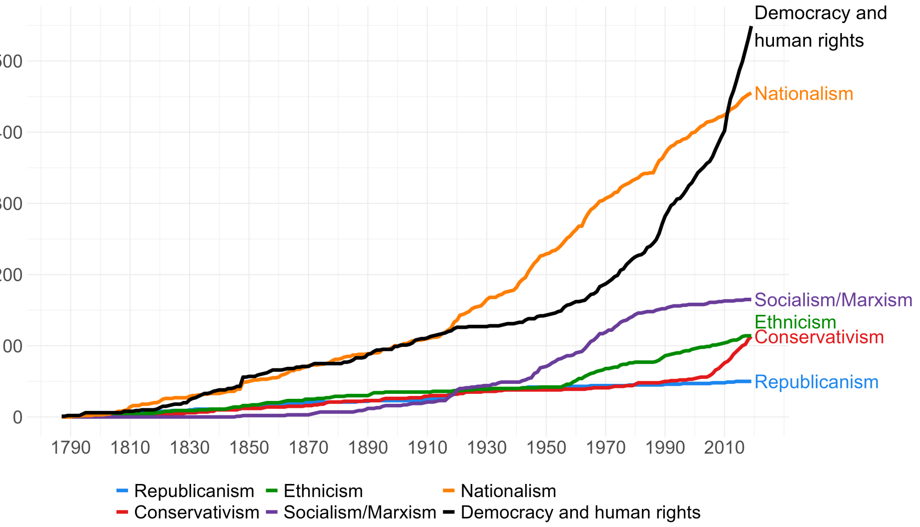

Figure B.7: Cumulative count of campaigns over time, categorized by ideology, excluding campaigns supporting the current regime or government.

Ideology share, opposition movements only

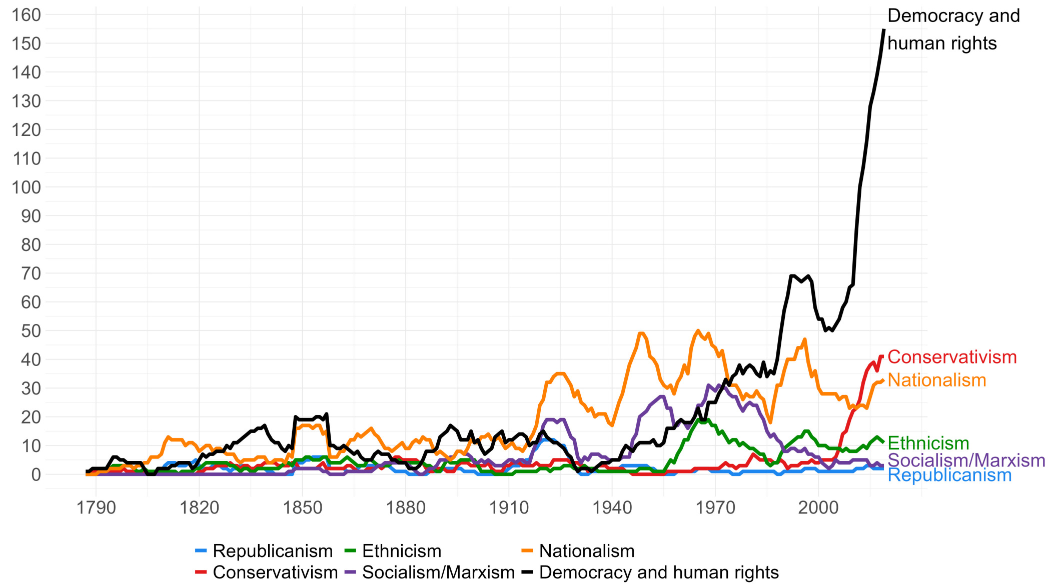

Figure B.8: Number of campaigns over time, categorized by ideology, sum of the past 10 year, excluding campaigns supporting the current regime or government.

Dominating social group, opposition movements only

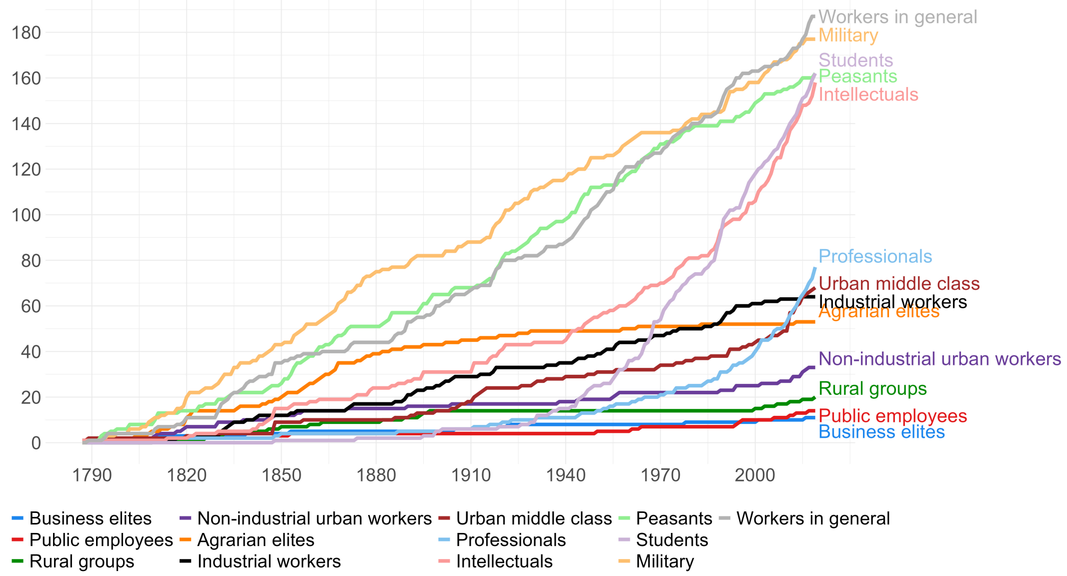

Figure B.9: Cumulative count of campaigns over time, by dominating social group. excluding campaigns supporting the current regime or government.

Organization and coordination, opposition movements only

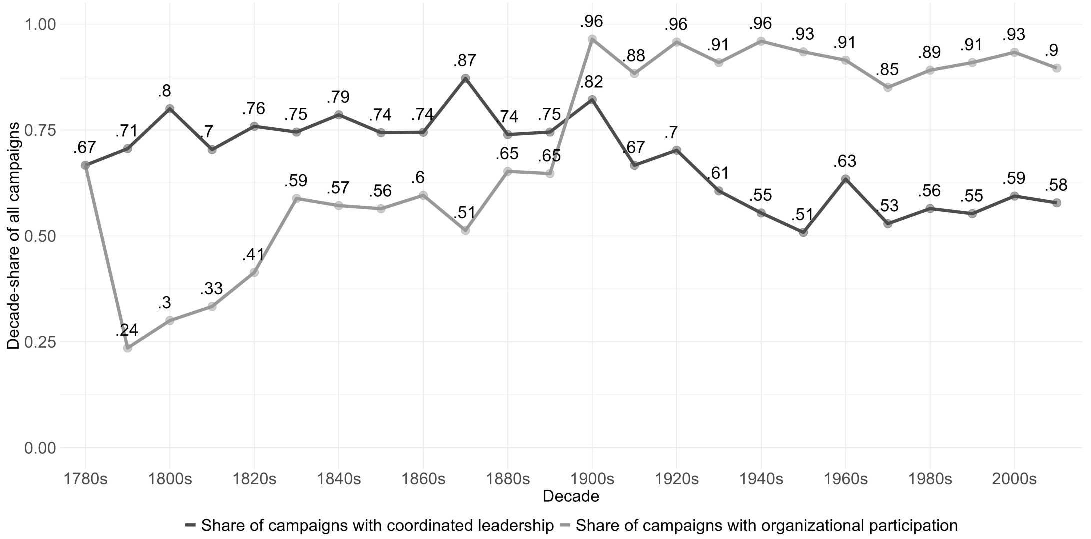

Figure B.10: Share of all campaigns by decade with party or civil society organization participation (light grey) and coordinated leadership (dark grey), excluding campaigns supporting the current regime or government.

C Additional PanelMatch models and pre-trends

C.1 Additional information to models presented in the main article

We here present the PanelMatch-results from the main article in tabular format, as well as the balance in pre-trends for all covariates in each model. We do not provide a full list of all treated units and corresponding control units, but this can be obtained from the replication material.

Additional information to results presented in Figure 7

Table C.1: ATT for violent vs. nonviolent protest movements on subsequent Polyarchy-scores

Time	Violent	Nonviolent
T+0	-0.0033	-0.0026
	(0.0044)	(0.0042)
T+1	-0.0017	0.016*
	(0.0065)	(0.0081)
T+2	4e-04	0.032*
	(0.0068)	(0.0105)
T+3	-0.002	0.0431*
	(0.007)	(0.0118)
T+4	-0.0037	0.0477*
	(0.0073)	(0.0121)
T+5	-0.004	0.0526*
	(0.0078)	(0.0126)
T+6	-0.0076	0.0607*
	(0.0083)	(0.0124)
T+7	-0.0079	0.0708*
	(0.0088)	(0.0137)
T+8	-0.0059	0.0694*
	(0.0101)	(0.0139)
T+9	-0.0019	0.0714*
	(0.0104)	(0.0138)
T+10	-0.0028	0.0764*
	(0.0113)	(0.0138)
N treated	197	199
Sum of control units	4916	5324

** p <0.05. Standard errors in parentheses*

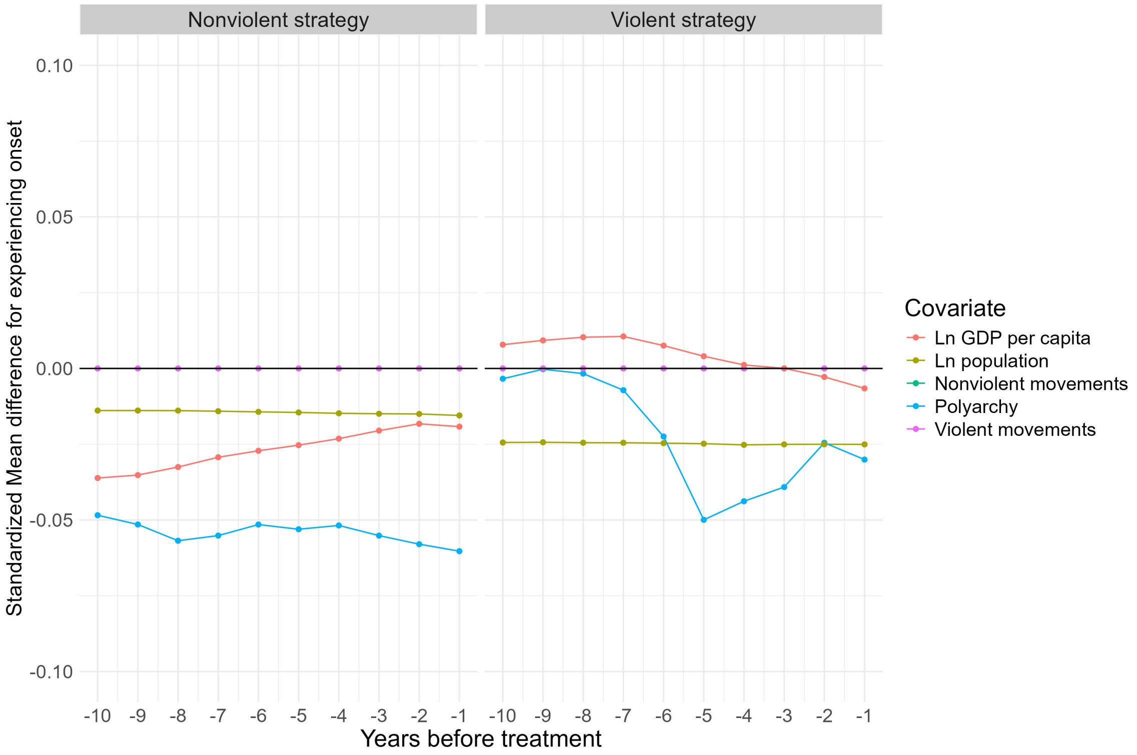

Figure C.1: Pre-trend balance for models in figure 7

C.1.1 Additional information to results presented in Figure 8

Table C.2: ATT for violent protest movements on subsequent Polyarchy- scores across different time periods

Time	Before 1900	After 1900	After 1945
T+0	0.0042	-0.0077	-0.0054
	(0.0053)	(0.0067)	(0.009)
T+1	0.0074	-0.006	-0.0107
	(0.0052)	(0.0104)	(0.0164)
T+2	0.0046	-5e-04	-0.0054
	(0.0046)	(0.0117)	(0.0173)
T+3	0.0072	-0.0064	-0.0084
	(0.0062)	(0.0123)	(0.0178)
T+4	0.0031	-0.0077	-0.0076
	(0.0077)	(0.0126)	(0.0182)
T+5	0.0048	-0.0094	-0.0059
	(0.0083)	(0.0138)	(0.0205)
T+6	-0.003	-0.0117	-0.0055
	(0.0071)	(0.0146)	(0.0196)
T+7	-0.0031	-0.0129	-0.0054
	(0.0063)	(0.0153)	(0.02)
T+8	-0.0081	-0.0074	7e-04
	(0.0052)	(0.0174)	(0.022)
T+9	-0.0073	-1e-04	0.009
	(0.0056)	(0.0181)	(0.0221)
T+10	-0.0129*	0.0018	0.009
	(0.0063)	(0.0196)	(0.0258)
N treated	73	108	63
Sum of control units	1332	3377	1875

** $p < 0.05$. Standard errors in parentheses*

Table C.3: ATT for nonviolent protest movements on subsequent Polyarchy- scores across different time periods

Time	Before 1900	After 1900	After 1945
T+0	-0.0063	-0.0023	9e-04
	(0.0137)	(0.0046)	(0.0051)
T+1	0.0163	0.0159	0.0218*
	(0.014)	(0.0089)	(0.0102)
T+2	0.0182	0.0341*	0.0457*
	(0.0137)	(0.0115)	(0.0133)
T+3	0.0228	0.0454*	0.0557*
	(0.0234)	(0.013)	(0.0154)
T+4	0.0141	0.0501*	0.0587*
	(0.03)	(0.0131)	(0.0155)
T+5	0.0124	0.0557*	0.0591*
	(0.0309)	(0.0134)	(0.016)
T+6	0.014	0.0653*	0.0653*
	(0.0319)	(0.0134)	(0.0163)
T+7	0.0111	0.0757*	0.0769*
	(0.0333)	(0.0149)	(0.0176)
T+8	0.0164	0.0732*	0.0739*
	(0.0313)	(0.0153)	(0.0177)
T+9	0.0227	0.0739*	0.0725*
	(0.0303)	(0.0151)	(0.0176)
T+10	0.0216	0.0795*	0.079*
	(0.0313)	(0.0152)	(0.0175)
N treated	15	173	140
Sum of control units	298	4883	3758

** p <0.05. Standard errors in parentheses*

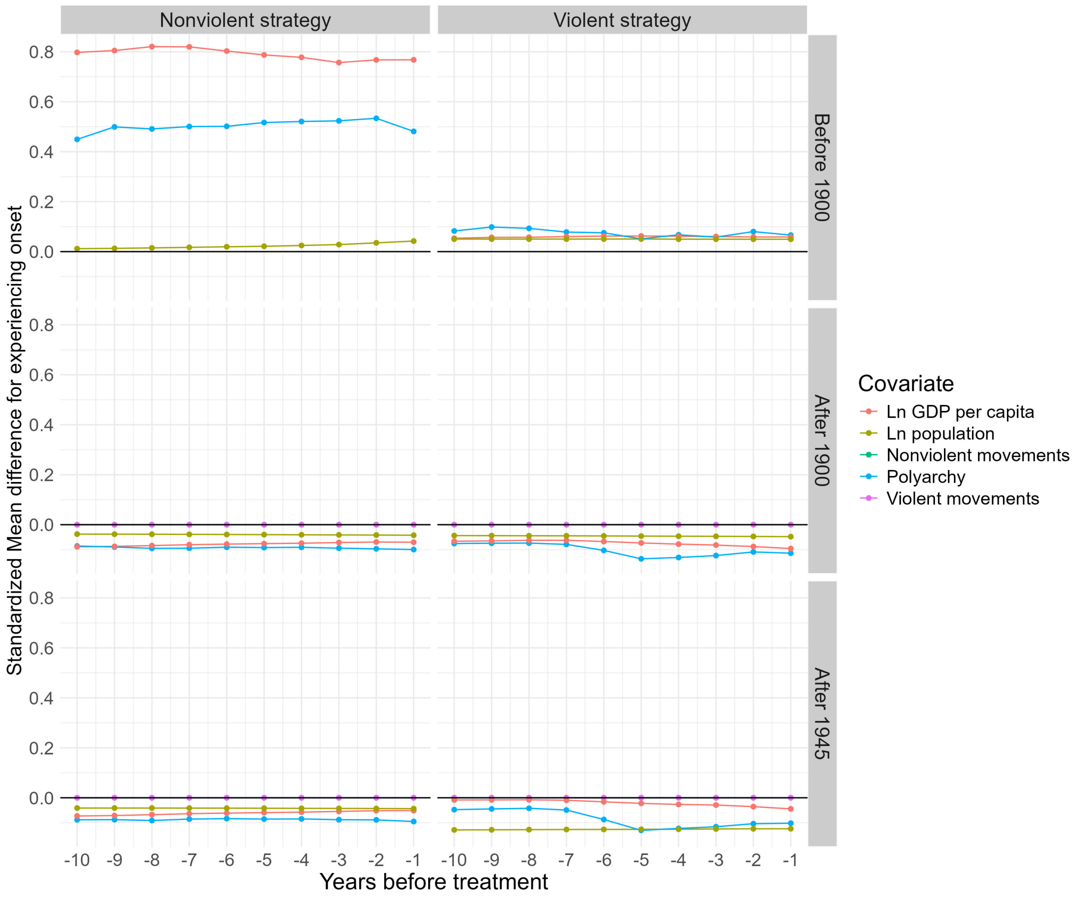

Figure C.2: Pre-trend balance for models in figure 8

C.2 Additional PanelMatch analyses

In this section, we present additional PanelMatch results (in tables) and analyses that are referred to, but not presented, in the main article. Specifically, we present estimates of the ATT of violent versus nonviolent campaigns across campaigns with different demands and different dominating social groups.

ATT for campaigns with different demands

Here we present the ATT for violent and nonviolent campaigns with different demands, estimated using the PanelMatch setup similar to our benchmark models, in a tabular format.

Table C.4: ATT for violent protest movements on subsequent Polyarchy- scores across different goals

Time	Autonomy	Secession	Remove gov	Remove regime	Institutional
T+0	8e-04	0.0031	-0.0047	-0.0036	0.0013
	(0.0049)	(0.0055)	(0.0034)	(0.0105)	(0.0067)
T+1	-0.0112	0.0067	-0.011	0.0095	-0.0029
	(0.013)	(0.0072)	(0.0063)	(0.0161)	(0.0068)
T+2	0.0021	0.0071	-0.0117	0.0158	0.0013
	(0.0252)	(0.0116)	(0.0073)	(0.016)	(0.0063)
T+3	-0.0089	0.001	-0.0219*	0.014	0.007
	(0.035)	(0.016)	(0.0098)	(0.0153)	(0.0095)
T+4	-0.0105	0.0017	-0.0229	0.0106	0.0076
	(0.0357)	(0.0167)	(0.0117)	(0.0153)	(0.0096)
T+5	-0.0526	-0.0044	-0.0244	0.0171	0.007
	(0.0437)	(0.0186)	(0.0128)	(0.0143)	(0.0126)
T+6	-0.0498	-0.0072	-0.0294*	0.0098	0.0045
	(0.0559)	(0.0198)	(0.0138)	(0.016)	(0.0131)
T+7	-0.0532	-0.0067	-0.028*	0.0059	0.0064
	(0.0563)	(0.0201)	(0.0139)	(0.018)	(0.0127)
T+8	-0.0702	-0.007	-0.0266	0.0071	0.0119
	(0.057)	(0.0205)	(0.0156)	(0.0221)	(0.0153)
T+9	-0.0425	-0.0092	-0.0215	0.011	0.0125
	(0.0667)	(0.0218)	(0.0165)	(0.0232)	(0.0158)
T+10	-0.031	-0.0125	-0.0319	0.0228	0.005
	(0.0709)	(0.0262)	(0.0213)	(0.0232)	(0.0181)
N treated	10	47	46	76	24
Sum of control units	255	1229	1076	2031	511

** p <0.05. Standard errors in parentheses*

Table C.5: ATT for nonviolent protest movements on subsequent Polyarchy- scores across different goals

Time	Autonomy	Secession	Remove gov	Remove regime	Institutional
T+0	0.0191	0.0344	-0.0171	-0.0015	-0.0016
	(0.0187)	(0.0498)	(0.0134)	(0.0079)	(0.0026)
T+1	0.0237	0.0708	0.0016	0.0139	0.0146
	(0.0497)	(0.07)	(0.0225)	(0.0148)	(0.0116)
T+2	0.0184	0.0738	0.0231	0.0416	0.0345*
	(0.0934)	(0.0704)	(0.0262)	(0.0251)	(0.0143)
T+3	0.103	0.087	0.0368	0.0499	0.0463*
	(0.098)	(0.07)	(0.0277)	(0.0333)	(0.0164)
T+4	0.1101	0.0933	0.0486	0.0498	0.0509*
	(0.1005)	(0.0707)	(0.0275)	(0.0353)	(0.0169)
T+5	0.1026	0.0955	0.0519	0.0651	0.0495*
	(0.1011)	(0.0713)	(0.0276)	(0.0348)	(0.017)
T+6	0.1018	0.0892	0.0512	0.0796*	0.062*
	(0.1028)	(0.0716)	(0.028)	(0.0331)	(0.0178)
T+7	0.0993	0.0974	0.0511	0.1048*	0.0721*
	(0.1041)	(0.0712)	(0.0288)	(0.0378)	(0.0191)
T+8	0.0819	0.0838	0.0369	0.1085*	0.072*
	(0.1068)	(0.0752)	(0.0284)	(0.0388)	(0.0196)
T+9	0.0599	0.1054	0.0247	0.1128*	0.0831*
	(0.109)	(0.0798)	(0.0289)	(0.0401)	(0.0194)
T+10	0.0508	0.0979	0.0325	0.1233*	0.0851*
	(0.1107)	(0.0791)	(0.0292)	(0.0408)	(0.02)
N treated	8	11	46	46	82
Sum of control units	192	193	1296	1575	2092

** p <0.05. Standard errors in parentheses*

ATT for campaigns with different ideologies

In this section, we present the ATT for violent and nonviolent campaigns with different ideologies, estimated by using the PanelMatch setup similar to our benchmark models.

Table C.6: ATT for violent protest movements on subsequent Polyarchy-scores across different ideologies

Time	Socialism and Marxism	Democracy and HR	Nationalism	Conservativism
T+0	0.02	0.014	-6e-04	-9e-04
	(0.0147)	(0.0165)	(0.0032)	(0.0263)
T+1	0.0486*	0.02	-0.0012	0.0066
	(0.0243)	(0.0228)	(0.0052)	(0.026)
T+2	0.0467	0.0127	-0.0047	0.0097
	(0.0259)	(0.0253)	(0.008)	(0.0313)
T+3	0.0308	0.0185	-0.016	8e-04
	(0.0256)	(0.0246)	(0.0107)	(0.0441)
T+4	0.0213	0.0102	-0.0176	-0.0086
	(0.023)	(0.0193)	(0.0107)	(0.0487)
T+5	0.0197	0.0129	-0.0215	-0.0161
	(0.0258)	(0.0213)	(0.0118)	(0.0514)
T+6	0.0219	0.0077	-0.0264	-0.0351
	(0.0276)	(0.02)	(0.0139)	(0.0615)
T+7	0.0255	0.0108	-0.032*	-0.054
	(0.0285)	(0.0197)	(0.0153)	(0.0776)
T+8	0.0271	0.0325	-0.0331*	-0.061
	(0.0297)	(0.023)	(0.0156)	(0.0768)
T+9	0.032	0.0312	-0.0238	-0.0614
	(0.031)	(0.0237)	(0.0162)	(0.0766)
T+10	0.0306	0.0346	-0.023	-0.0857
	(0.0338)	(0.0241)	(0.0205)	(0.0686)
N treated	37	28	79	11
Sum of control units	1137	593	2199	262

** p <0.05. Standard errors in parentheses*

Table C.7: ATT for nonviolent protest movements on subsequent Polyarchy- scores across different ideologies

Time	Socialism and Marxism	Democracy and HR	Nationalism	Conservativism
T+0	0.0029	-1e-04	-0.0235	-0.0115
	(0.0086)	(0.0038)	(0.0158)	(0.0181)
T+1	0.0071	0.029*	-0.0252	-0.0146
	(0.0115)	(0.0116)	(0.0255)	(0.0234)
T+2	0.0197	0.0585*	-0.031	-0.0133
	(0.0171)	(0.015)	(0.0346)	(0.0236)
T+3	0.049	0.073*	-0.0182	-0.0306
	(0.0268)	(0.0169)	(0.0466)	(0.0296)
T+4	0.0522	0.0747*	-0.0124	-0.0235
	(0.0322)	(0.0175)	(0.0458)	(0.0322)
T+5	0.0568	0.0724*	0.0305	0.0156
	(0.032)	(0.0182)	(0.0417)	(0.0441)
T+6	0.0697*	0.0793*	0.0498	0.0101
	(0.035)	(0.0184)	(0.0353)	(0.0448)
T+7	0.0729*	0.0965*	0.0537	0.0042
	(0.0358)	(0.0201)	(0.036)	(0.0453)
T+8	0.0708*	0.0985*	0.0489	0.0103
	(0.0357)	(0.0209)	(0.0379)	(0.0452)
T+9	0.0731	0.1031*	0.0498	0.0091
	(0.0372)	(0.0213)	(0.0408)	(0.0485)
T+10	0.0677	0.1094*	0.047	0.0146
	(0.0365)	(0.0219)	(0.0402)	(0.0519)
N treated	26	116	27	11
Sum of control units	754	3125	873	340

** p <0.05. Standard errors in parentheses*

ATT for campaigns with different dominating social group

In this section, we present the ATT for violent and nonviolent campaigns with different dominating social groups, estimated by using the PanelMatch setup similar to our benchmark models.

Table C.8: ATT for violent protest movements on subsequent Polyarchy- scores across different social groups

Time	Workers	Students	Military	Peasants	Urban mid. class	Intellectuals
T+0	0.0286	-0.0364	-9e-04	9e-04	0.0061	-0.0168
	(0.0185)	(0.0335)	(0.0075)	(0.0113)	(0.0479)	(0.0093)
T+1	0.0506	-0.0697	0.0099	-9e-04	0.0224	-0.0232
	(0.0296)	(0.055)	(0.0144)	(0.0175)	(0.0536)	(0.013)
T+2	0.0408	-0.0966	0.0089	0.0023	0.0275	-0.0312
	(0.0309)	(0.0771)	(0.016)	(0.0183)	(0.0552)	(0.0176)
T+3	0.0294	-0.0923	0.0054	4e-04	0.008	-0.0283
	(0.0309)	(0.0573)	(0.0174)	(0.0187)	(0.0611)	(0.018)
T+4	0.0154	-0.0757	0.0046	0.0012	-0.0339	-0.0189
	(0.0296)	(0.0418)	(0.0192)	(0.0185)	(0.0666)	(0.0217)
T+5	0.0151	-0.0512	0.0126	0.0103	-0.0533	-0.0165
	(0.0371)	(0.043)	(0.0194)	(0.0195)	(0.0808)	(0.0253)
T+6	0.0129	-0.0463	-0.0036	0.0077	-0.0664	-0.0194
	(0.0424)	(0.0449)	(0.0193)	(0.0202)	(0.0925)	(0.0306)
T+7	0.0118	-0.0422	-0.015	0.0066	-0.0672	-0.0129
	(0.0427)	(0.0417)	(0.0226)	(0.0208)	(0.0935)	(0.0339)
T+8	0.0177	0.0662	-0.0231	0.0039	-0.0754	0.0185
	(0.0427)	(0.0622)	(0.023)	(0.0216)	(0.0955)	(0.0501)
T+9	0.0235	0.077	-0.0161	0.0114	-0.0706	0.0225
	(0.0425)	(0.0748)	(0.0231)	(0.0208)	(0.0984)	(0.0554)
T+10	0.0366	0.0949	-0.0161	0.0137	-0.0579	0.0185
	(0.0439)	(0.0791)	(0.0233)	(0.0187)	(0.0983)	(0.0569)
N treated	23	6	37	43	10	21
Sum of control units	679	210	955	1042	185	446

** $p < 0.05$. Standard errors in parentheses*

Table C.9: ATT for nonviolent protest movements on subsequent Polyarchy- scores across different social groups

Time	Workers	Students	Military	Peasants	Urban mid. class	Intellectuals
T+0	0.0018	2e-04	-0.007	-0.0101*	-0.0055	0.003
	(0.0054)	(0.005)	(0.0668)	(0.0045)	(0.0135)	(0.0038)
T+1	0.0382	0.0232*	0.0367	-0.0075	0.0229	0.0214
	(0.0199)	(0.0116)	(0.1302)	(0.0095)	(0.0306)	(0.0126)
T+2	0.0592*	0.0642*	0.0188	-0.0034	0.0217	0.0363
	(0.0275)	(0.021)	(0.1555)	(0.0152)	(0.0316)	(0.0224)
T+3	0.0731*	0.0769*	0.0182	-0.0046	0.0326	0.0394
	(0.0315)	(0.0256)	(0.1583)	(0.0248)	(0.032)	(0.025)
T+4	0.0822*	0.0766*	0.0186	-0.026	0.0167	0.045
	(0.0332)	(0.0262)	(0.1577)	(0.0379)	(0.0474)	(0.0259)
T+5	0.0878*	0.0795*	0.0302	-0.0328	0.0028	0.0398
	(0.0364)	(0.0269)	(0.1417)	(0.0374)	(0.0612)	(0.0256)
T+6	0.0932*	0.0867*	0.1202	-0.0281	0.0117	0.0486
	(0.0381)	(0.0277)	(0.0904)	(0.0385)	(0.0607)	(0.0258)
T+7	0.1234*	0.0902*	0.1144	-0.006	0.0138	0.0556*
	(0.0416)	(0.0288)	(0.0912)	(0.0413)	(0.0616)	(0.0261)
T+8	0.1216*	0.093*	0.1176	-0.0052	0.0183	0.061*
	(0.0419)	(0.0292)	(0.0916)	(0.0418)	(0.0632)	(0.0264)
T+9	0.1342*	0.0921*	0.1156	0.0059	0.022	0.082*
	(0.0423)	(0.0294)	(0.0924)	(0.0458)	(0.0605)	(0.0267)
T+10	0.1362*	0.0851*	0.1067	0.0237	0.0187	0.0948*
	(0.0435)	(0.0305)	(0.0944)	(0.0466)	(0.0602)	(0.0275)
N treated	39	43	6	14	8	31
Sum of control units	1082	1395	165	415	131	743

** p <0.05. Standard errors in parentheses*

Including flanks in violent and nonviolent strategies

We here present the ATT for violent and nonviolent campaigns, but counting violent movements with nonviolent flanks as nonviolent movements, and nonviolent movements with violent flanks as violent movements. When matching on the presence of other campaigns, we similarly match on violent campaigns measured with violent flanks in predominantly nonviolent movements included, and nonviolent campaigns measured with nonviolent flanks in predominantly violent movements included. Otherwise, the PanelMatch setup is similar to our benchmark models.

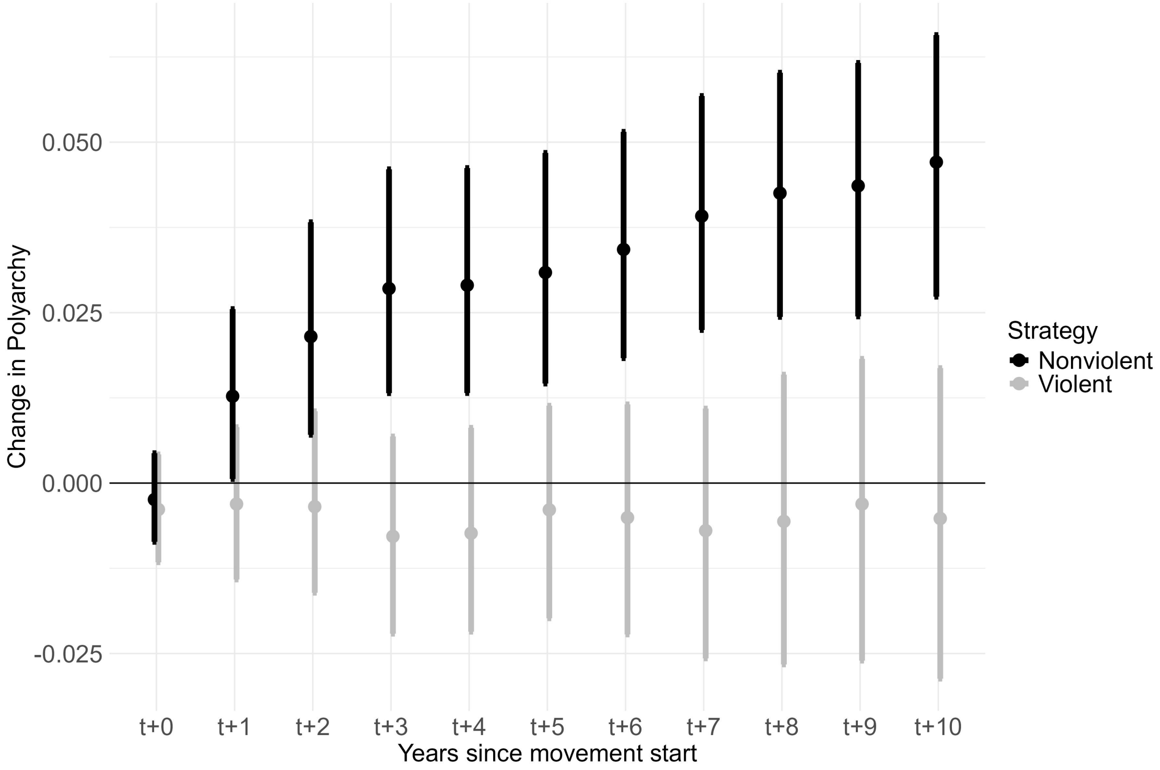

Figure C.3: ATT for violent vs. nonviolent protest movements on subsequent Polyarchy-scores, including violent movements with nonviolent flanks as nonviolent movements, and nonviolent movements with violent flanks as violent movements. Points indicate the point-estimate for the ATT of the respective strategy on Polyarchy (Y-axis) in 0–10 year (X-axis) after the first year of the movement. The bars represent the 95% confidence interval for the point-estimate.

ATT for violent and nonviolent campaigns including additional controls for instability

Here we present ATT results for violent and nonviolent campaigns similar to the main results presented in Figure 7, but controlling for whether the polity was an independent state, had domestic autonomy (extent of territorial control), the presence of ongoing civil war, and the number of successful coups in the polity's history, in addition to the benchmark controls of Ln population and Ln GDP per capita. Independence is a binary

Table C.10: ATT for violent vs. nonviolent protest movements on subsequent Polyarchy-scores

Time	Violent	Nonviolent
T+0	-0.0039	-0.0024
	(0.004)	(0.0035)
T+1	-0.0031	0.0127
	(0.0061)	(0.0065)
T+2	-0.0035	0.0215*
	(0.0068)	(0.0082)
T+3	-0.0078	0.0285*
	(0.0076)	(0.0088)
T+4	-0.0074	0.029*
	(0.0078)	(0.0088)
T+5	-0.0039	0.0309*
	(0.0082)	(0.009)
T+6	-0.0051	0.0343*
	(0.0087)	(0.0087)
T+7	-0.007	0.0392*
	(0.0094)	(0.0085)
T+8	-0.0056	0.0425*
	(0.0109)	(0.0089)
T+9	-0.0031	0.0436*
	(0.0112)	(0.0092)
T+10	-0.0052	0.0471*
	(0.0118)	(0.0098)
N treated	208	299
Sum of control units	5493	10852

** $p < 0.05$. Standard errors in parentheses*

variable from V-Dem (Coppedge, 2022). Occupation is a scalar variable from V-Dem measuring the government's territorial control within the country's borders. It differs from independence by including additional situations that result in autonomy losses, such as foreign occupation of parts of the territory. Ongoing civil war is defined as as any intra-state war causing at least 1,000 battle deaths, with data taken from Haber and Menaldo (2011). The count variable of successful military coups is derived from the "Political Institutions and Political Events" (PIPE) dataset (Przeworski et al., 2013). The inclusion of these variables slightly heightens the uncertainty of the ATT estimates but does not substantially alter the main results.

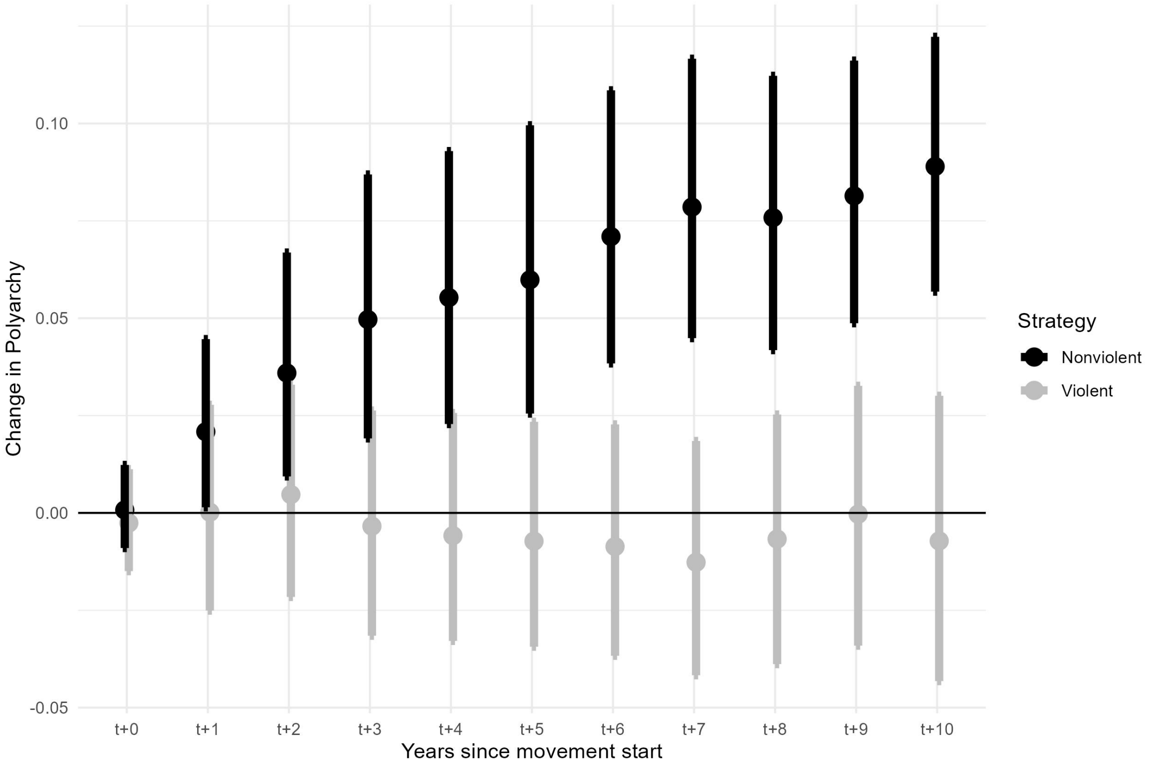

Figure C.4: ATT for violent vs. nonviolent protest opposition movements on subsequent Polyarchy-scores. Points indicate the point-estimate for the ATT of violent (grey) and nonviolent (black) movements on Polyarchy (Y-axis) in 0–10 year (X-axis) after the first year of the movement. Bars indicate the 95% confidence interval for these estimates. In addition to population and GDP p.c., these models also account for whether the polity was independent, occupied, had an ongoing civil war, and the number of successful coups so far in the polity's history.

D Comparison between OMG and existing datasets

In Figure D.1, we compare the number of ongoing campaigns recorded for each country-year in OMG and in NAVCO 2.1. In making these comparisons, we only include the countries coded by both datasets between 1945 and 2013. In total, 355 NAVCO campaigns are coded for this sample, and 651 OMG campaigns (813 campaign-phases). 103 countries that have at least 1 movement throughout the timeline in either dataset are coded by both datasets.

Furthermore, our comparison is restricted to country-years with at least one ongoing campaign in either dataset. Including years without campaigns inflates the correlations, as most country-years do not have active campaigns. OMG generally records a higher number of active campaigns than NAVCO: 66% of all country-years with any campaign report more ongoing campaigns in OMG, while 14% have more campaigns recorded in NAVCO. The remaining 20% of country-years have equal numbers of campaigns, placed on the diagonal in Figure D. Some of the deviations between the two datasets may stem from measurement errors, or more specifically unintentional missingness due to lacking identification of campaigns by coders. Yet, we highlight that much of the deviation can be explained by differences in operational rules between the two datasets.

The relatively high number of OMG campaigns is, in large part, due to the inclusion of "semi-maximalist" campaigns–those that, while not explicitly demanding regime change, leader change, or independence, still call for more than policy changes. These campaigns typically seek specific institutional reforms or limited territorial autonomy. One example is the Nigerien women's movement in Niamey. On May 11. 1991, more than 1000 women marched from the national assembly to the prime minister's office and occupied the ministry of external affairs. The movement demanded equal representation in Nigerien politics and the National Sovereign Conference, but also carried signs reading, for example, "Stop Injustice!" and "Equal Rights!".

Additionally, OMG includes one-off events lasting a day or less, provided they meet other definitional criteria. In contrast, NAVCO 2.1 requires campaigns to last for at least two weeks to be included in the dataset. Examples of such one-off events include the October 25 1965 student protests in Kabul, Afghanistan, during which more than 1,000 students mobilized to demand the government's resignation. Protesters marched from Kabul University, blocking security forces en route to parliament. The demonstration escalated when security forces intervened, resulting in violent clashes and three fatalities.

Another example is the Egyptian women's liberation movement of 1952, during which Doria Shafiq led a march of more than 1,500 women to storm the gates of the parliament to demand the right to vote. Today, the significance of including or excluding one-off events is perhaps best illustrated by the "January 6. Insurrection" in USA, which would have been included in OMG if the coding extended to this date, but excluded (to the best of our understanding) from NAVCO 2.1, given its definitional criteria.[36]

Third, as discussed in the paper, OMG also includes pro-government and pro-regime

[36]Excluding campaigns lasting less than two weeks may introduce systematic selection bias, as these campaigns are likely to over-represent two "types" of campaigns. First, rapid successes where significant political or social change occurs without much resistance from a crumbling regime; and second, campaigns that are swiftly suppressed by immediate and unified government repression, resulting in their termination in less than two weeks.

campaigns in the post-1900 period, whereas NAVCO focuses exclusively on opposition campaigns. Out of the 1431 of campaign-phases in the OMG dataset for the post-1900 period, 229 (16%) have such "pro-campaigns" demands, and 69 (4.8%) are anti-liberal.

On the other hand, two types of campaigns are included in NAVCO 2.1, but not in OMG. First, a set of smaller countries (population less than 1 million) are included in NAVCO, but not in OMG. This follows, in part, from OMG's dependence on V-Dem having coded the country unit. V-Dem provides definitions of, but does not code, several smaller polities such as Bahamas, Liechtenstein, or Tonga. Other smaller units are coded by V-Dem, but not (at least in this version of) OMG, simply due to resources constraints. Note, however, that these deviations are not reflected in Figure D.1, as it only includes countries present in both datasets.

Second, OMG requires that the location of the campaign and the polity-target align. This criterion excludes, for example, the 1991 "Third Hutu Uprising" in Burundi, which is included in NAVCO 2.1. The exclusion in OMG is due to the fact that the activities – while directed at the Burundian government – were launched from neighboring countries, with no evidence of sufficient mobilization within Burundi itself. As a result, the Third Hutu Uprising is listed only as a candidate campaign in OMG, but is not included in the final dataset.

Many differences, however, stem from judgment-based discrepancies. In this regard, it is worth noting that OMG – being coded later in time – had the benefit of NAVCO-coded campaigns being available, reducing the probability of coders unintentionally missing these campaigns. Yet, OMG coders still followed the protocol laid out in the paper, and treated NAVCO-coded campaigns as candidate campaigns, and searched for evidence on whether or not each candidate fulfilled the different definitional criteria. For instance, and unlike NAVCO, OMG found no evidence that the 1989 Tripuri secessionist movement in India mobilized more than 1,000 individuals. Consequently, the campaign was included only in OMG's candidate list and excluded from the final dataset. This omission results in OMG recording six ongoing campaigns in India during most of the 1990s, compared to NAVCO's seven. This discrepancy accounts for all instances in Figure D.1 where NAVCO records seven campaigns within a country-year, while OMG records six. The example also illustrates that the correlations shown in the figure are influenced by that, for every omitted or included campaign, all years during which the campaign is active are affected.

We conduct a similar comparison between OMG and the Revolutionary Episodes dataset (RED) (Beissinger, 2022a), and the overall pattern is presented in Figure D.2. RED employs a more restrictive definition of what constitutes a maximalist demand than OMG and NAVCO. Elaborating on the difference with NAVCO, in Appendix 1, Beissinger (2022a, p. 439) writes that "a closer examination reveals that a large proportion of the "maximalist" campaigns in the NAVCO data involved instances of resistance to foreign invasions or foreign occupations, were irredentist in character, or were quasi-revolutionary episodes, reformist campaigns, or non-revolutionary sieges of government as outlined by my definitions in chapter 1."

The comparison is restricted to countries covered by both datasets for the 1900–2013 period, encompassing 305 RED campaigns and 927 OMG campaigns (1187 campaign-phases) in a total of 111 countries. In Figure D.2, we only compare the country-years in which at least one dataset has recorded at least one campaign. As we should expect from the definitional rules and the inclusion of non-maximalist campaigns, OMG records

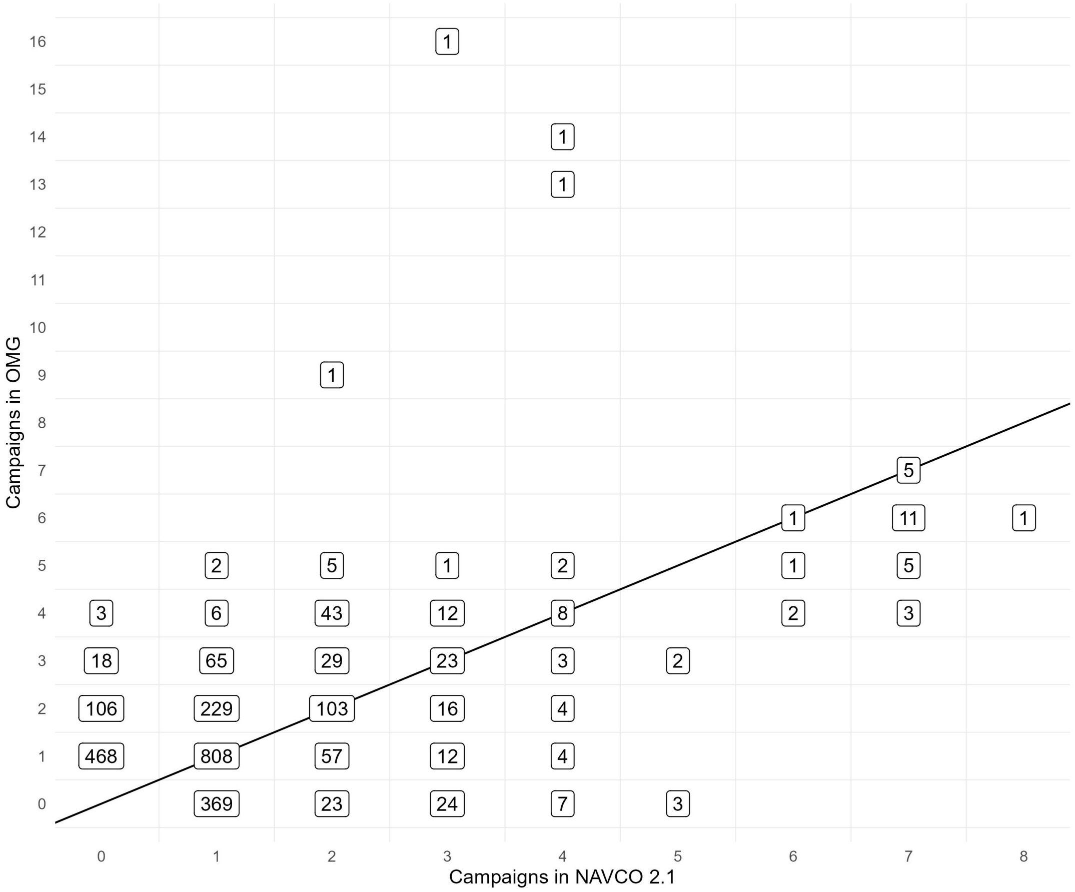

Figure D.1: Correlation between OMG and NAVCO 2.1 in the number of ongoing campaigns in each country-year, for countries present in both datasets, 1945-2013. The black diagonal line indicates the cells where the two datasets have an equal number of ongoing campaigns. The number in each cells is the count of country-years with the respective combination of ongoing campaigns in OMG (Y-axis) and NAVCO (X-axis).

more ongoing campaigns than RED: 74% of country-years have more ongoing campaigns in OMG than RED, while 11% of country-years have more campaigns in RED. The remaining 15% of country-years records the same number of campaigns in both datasets.

Still, some campaigns are included in RED but not in OMG, primarily due to differences in judgment. For example, the Mon uprising in Burma/Myanmar is included in RED, but OMG coders did not find sufficient evidence that it met the 1,000-participant threshold. Such judgment-based differences are an inherent challenge to the complex task of coding mass mobilization. However, they underscore the importance of diverse and independent efforts to systematically map mass movements, as they, at the very least (without further digging into the sources and validation and adjudication efforts) indicate campaigns that might either be omitted or included in one of the two datasets due to measurement error. These parallel efforts are, in extension, essential to testing whether relationships observed when using one dataset hold when using others, and are thus robust to this kind of potential measurement error inducing mistaken inclusion or omission of campaigns.

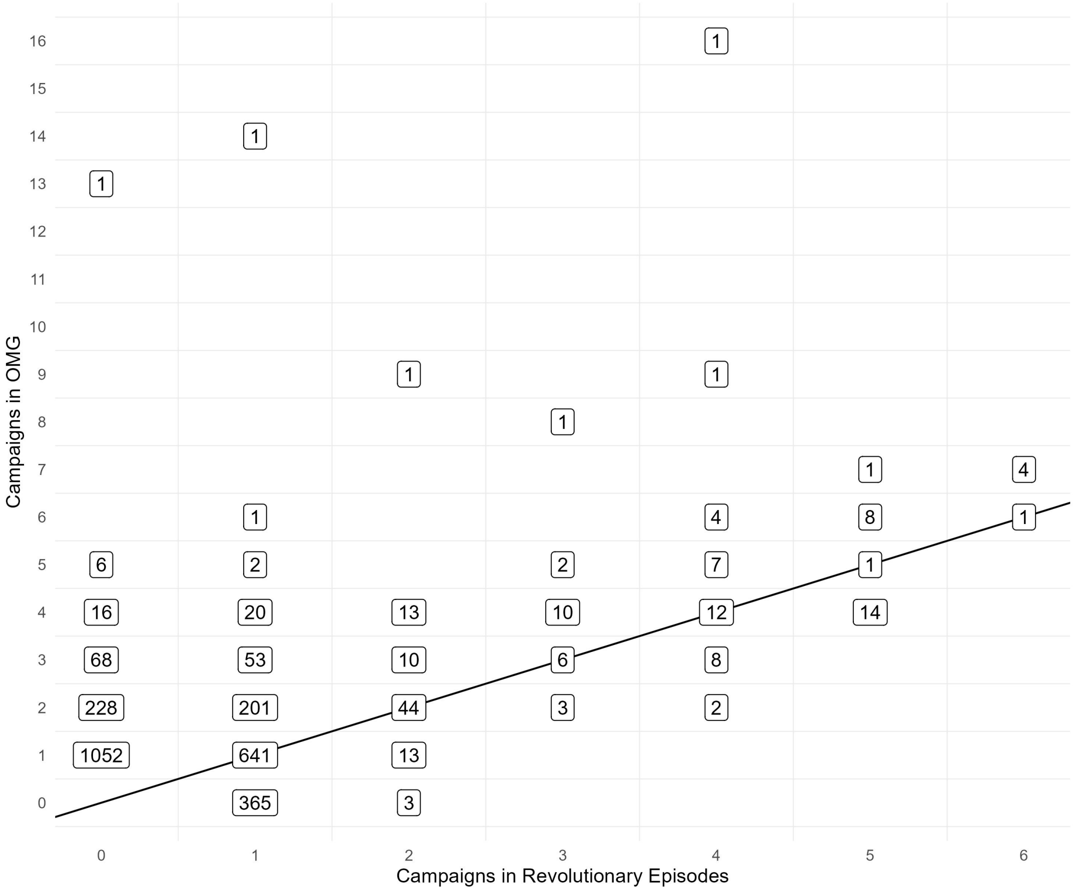

Figure D.2: Correlation between OMG and Revolutionary Episodes in the number of ongoing campaigns in each country-year, for countries present in both datasets, 1900-2014. The black diagonal line indicates the cells where the two datasets have an equal number of ongoing campaigns. The number in each cells is the count of country-years with the respective combination of ongoing campaigns in OMG (Y-axis) and RED (X-axis).

E Inter-coder reliability tests

To assess inter-coder reliability, some countries were coded by more than one coder. Yet, inter-coder reliability tests were not very extensive, as the team prioritized to spend resources and efforts on routines and measures that would reduce unsystematic measurement errors and ensure consistent coding across cases. As detailed in the paper, all coding processes were supervised to enhance reliability and ensure that ambiguous cases were consistently resolved. As expected, the frequency of supervisory comments gradually diminished with time, as coders gained proficiency and experience. This strategy, in other words, improved inter-coder reliability, at least conditional on the coders having undergone the training schemes and participating in the communication set-up to improve within-dataset consistency. Similarly, our double-coding efforts adhered to this strategy, and pertain to coders working under supervision. Notably, double-coding was conducted during the later stages of the process, ensuring that the reliability measures presented accurately reflect the consistency and quality of the final dataset, while they certainly underestimate the inter-coder reliability that would hypothetically obtain in a situation where two fresh coders started coding the same country without prior training.

With these caveats in mind, we used accuracy between pairs of coders as the primary metric to evaluate the level of inter-coder reliability. In total, 28 campaigns were subjected to double-coding. Accuracy is therefore the share of 28 campaigns where 2 coders agreed on the coding of the respective variable. The accuracy scores between pairs of coders are presented in Figure E.1. Overall, accuracy levels were high, with the mean accuracy for each group of variables exceeding 0.85. The group of variables with the lowest inter-coder reliability was the participation variables for different social groups, with "Intellectuals" and "Business elites" exhibiting accuracy scores as low as 0.71. While this is notably lower than other groups, it still represents a relatively high level of inter-coder reliability, given the relatively fine-grained groups included in the scheme, the fuzzy borders between them, and the low threshold for coding social group participation (i.e., identification of sources mentioning that at least one member of the social group participated, meaning that differences in coder skills at finding relevant sources or sheer luck may play some role). Overall, these tests do thus indicate that OMG is characterized by a high degree of internal consistency, and a low degree of unsystematic measurement error considering the complex phenomena being coded.

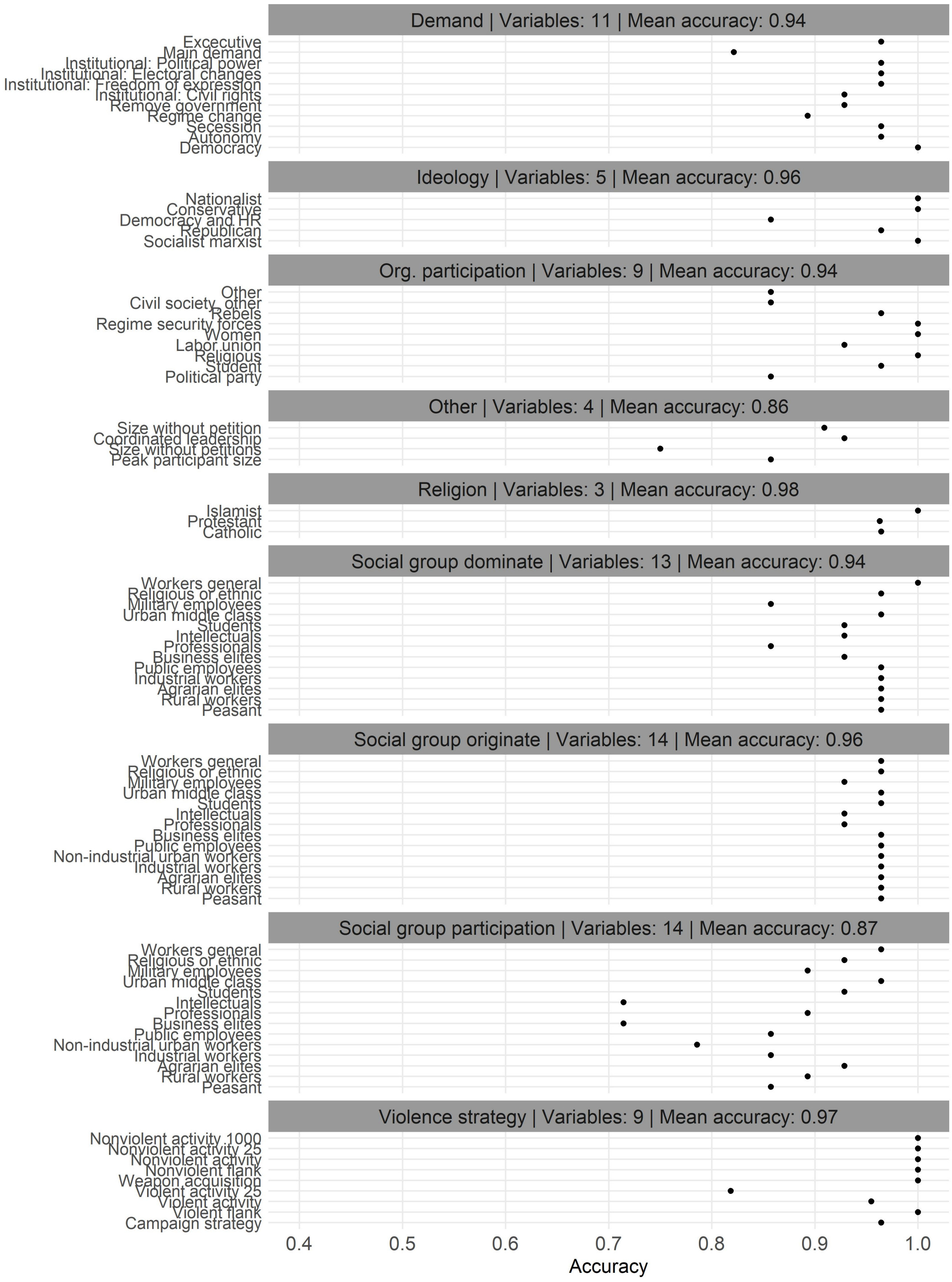

Figure E.1: Intercoder reliability, measured with accuracy, across variables.
